# Carbon Nanotubes as Excellent Adjuvants for Anticancer Therapeutics and Cancer Diagnosis: A Plethora of Laboratory Studies Versus Few Clinical Trials

**DOI:** 10.3390/cells14141052

**Published:** 2025-07-09

**Authors:** Silvana Alfei, Caterina Reggio, Guendalina Zuccari

**Affiliations:** 1 Department of Pharmacy (DIFAR), University of Genoa, Viale Cembrano, 4, 16148 Genoa, Italy; 2 Laboratory of Experimental Therapies in Oncology, IRCCS Istituto Giannina Gaslini, Via Gdg. Gaslini 5, 16147 Genoa, Italy; caterinareggio@gaslini.org

**Keywords:** carbon nanotubes (CNTs), CNT-improved anticancer therapies, CNT-improved cancer diagnosis, photothermal therapy (PTT), photodynamic therapy (PDT), gene therapy (GT), immunotherapy (IT)

## Abstract

Encouraging discoveries and excellent advances in the fight against cancer have led to innovative therapies such as photothermal therapy (PTT), photodynamic therapy (PDT), drug targeting (DT), gene therapy (GT), immunotherapy (IT), and therapies that combine these treatments with conventional chemotherapy (CT). Furthermore, 2,041,910 new cancer cases and 618,120 cancer deaths have been estimated in the United States for the year 2025. The low survival rate (<50%) and poor prognosis of several cancers, despite aggressive treatments, are due to therapy-induced secondary tumorigenesis and the emergence of drug resistance. Moreover, serious adverse effects and/or great pain usually arise during treatments and/or in survivors, thus lowering the overall effectiveness of these cures. Although prevention is of paramount importance, novel anticancer approaches are urgently needed to address these issues. In the field of anticancer nanomedicine, carbon nanotubes (CNTs) could be of exceptional help due to their intrinsic, unprecedented features, easy functionalization, and large surface area, allowing excellent drug loading. CNTs can serve as drug carriers and as ingredients to engineer multifunctional platforms associated with diverse treatments for both anticancer therapy and diagnosis. The present review debates the most relevant advancements about the adjuvant role that CNTs could have in cancer diagnosis and therapy if associated with PTT, PDT, DT, GT, CT, and IT. Numerous sensing strategies utilising various CNT-based sensors for cancer diagnosis have been discussed in detail, never forgetting the still not fully clarified toxicological aspects that may derive from their extensive use. The unsolved challenges that still hamper the possible translation of CNT-based material in clinics, including regulatory hurdles, have been discussed to push scientists to focus on the development of advanced synthetic and purification work-up procedures, thus achieving more perfect CNTs for their safer real-life clinical use.

## 1. Introduction: Carbon Nanotubes in Cancer Therapy

**Figure 1:**
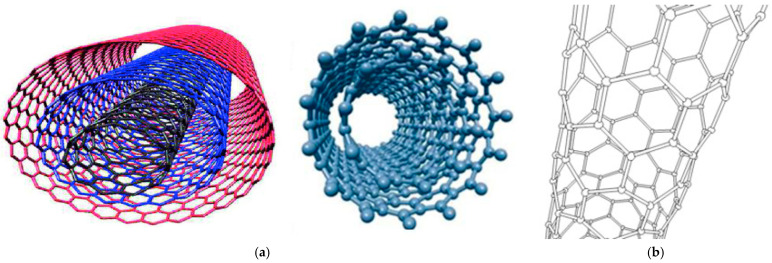
(**a**) Structures of two types of MWCNTs. On the left side of panel (**a**) there is the MWCNTs Russian Doll model [57], while the MWCNTs Parchment model is observable on the right side [58]. (**b**) Structure of a SWCNT. The left image in panel (**a**) has been reproduced by an article available under the Creative Commons CC-BY-NC-ND license, which permits non-commercial use of the work as published, while the right image in panel (**a**) and that in panel (**b**) are by an unknown author and are licensed under CC BY-SA 3.0, which does not require permissions.

Cancer is considered the nastiest disorder of the past decades, whose rate of mortality is at worrying levels [1]. It represents the major societal, public health, and economic problems in our century. Globally, 20 million new cases of cancer, including non-melanoma skin cancers (NMSCs), were observed in the year 2022, which were responsible for 9.7 million deaths [2]. Only in the United States, 2,041,910 new cancer cases and 618,120 cancer deaths have been estimated for this year (2025). Demographics-based predictions indicate that the number of new cases of cancer will reach 35 million worldwide by 2050. Cancer represents a notable obstacle to life expectancy, causing substantial societal and macroeconomic costs, which can depend on cancer types, geography, and gender [3]. This detrimental condition is often characterised by an uncontrolled development of cancer cells without inhibition, which invasively spread to other organs and tissues in the whole body [4]. As a direct consequence, the physiological functions of normal cells are impaired, and the health and quality of life of the affected individuals are totally compromised [5,6]. Investments in prevention, mainly regarding the attempt to limit/reduce the key risk factors for cancer, such as smoking, overweight, obesity, and infection, could avert millions of future cancer diagnoses and save many lives worldwide, leading to a massive economic and societal improvement in countries over the approaching years [7]. Meanwhile, the current state of cancer therapy has greatly improved owing to the rapid development of treatment modalities, such as surgery, chemotherapy, radiotherapy, endocrine therapy, immunotherapy, phototherapy, and gene therapy. Unfortunately, these promising therapeutic strategies still have to face numerous obstacles that limit their widespread use, particularly for the treatment of metastatic cancer and cancer cells that have acquired resistance [8,9]. In this scenario, consisting of a severe and lethal disease currently difficult to treat and a limited or poorly efficient arsenal to limit its global damage, new strategies to counteract cancer are urgently needed. In the recent year, bio nanoparticles (NPs), including single, centre/shell, and multi-material composite nanomaterials, in terms of inorganic/organic, inorganic/inorganic, organic/organic, and organic/inorganic composites [10,11,12,13,14,15,16,17,18,19], have become a cutting-edge cancer treatment option [20,21,22,23]. Different types of nanomaterials (NMs) have been experimented with to treat many diseases, including various types of cancer [24,25,26,27,28,29,30], demonstrating high efficiency as drug delivery systems (DDSs). By means of NMs, target anticancer treatments can be realised, thus enhancing the antitumour effects of traditional chemotherapeutics while reducing their side effects and toxicity to normal cells [28,31,32,33,34,35,36,37,38,39,40,41,42]. Additionally, due to their nonpareil physiochemical and structural characteristics, NMs have shown great potential also in the early diagnosis of cancer and imaging [43,44,45,46]. Collectively, non-targeting and targeting nanomaterials, simple nanosystems, and complex nanoplatforms were developed in the last decades, thanks to nanotechnology and nanomedicine, which were applied as single therapeutic strategies or in combined therapy. Several bioactive nanoparticles (NPs), including liposomes, albumin, and polymeric micelles, for cancer treatment have already been approved for clinical use and are already on the market [47,48]. Such NPs can rapidly cross the human biological barriers and accumulate at the tumour site [49,50,51], where they can incessantly release their drug content to maintain the appropriate blood concentration of the drug despite low dosage [52,53]. Among nanomaterials, carbon nanotubes (CNTs), discovered in 1991 by Ijima [54], belong to the fullerene (carbon allotropes and C60) and graphene family. In their pristine form, they are made of sp2-bonded carbon atoms, which confer on them a structure encompassing planar graphite sheets, which roll up forming tubes with a diameter of a few nanometers and a variable length, decidedly greater in size (microns) than the diameters [55]. As reported, CNTs comprise multiwalled carbon nanotubes (MWCNTs), which contain numerous concentric tubes sharing a common axis (Figure 1a), and single-walled carbon nanotubes (SWCNTs), which are made of a single graphene sheet ‘rolled’ into a tube (Figure 1b) [55,56]. Also, two models of MWCNTs are reported, including the Russian Doll model, in which sheets of graphite are arranged in concentric SWCNT cylinders (Figure 1a, left), and the Parchment model, in which a single sheet of graphite is rolled in around itself, resembling a scroll of parchment or a rolled-up newspaper (Figure 1a, right).

Different types of CNTs can exist depending on their length, internal diameter, chirality, or rotational conformations [56]. CNTs collect in their unique structure several nonpareil properties, such as a cylindric-like shape, great surface area, very small diameters and different lengths, low density, excellent chemical stability, mechanical resistance, thermal conductivity, photoluminescence, transparency, constructional durability, and remarkable electrical conductivity [59,60]. Additionally, when applied in medicinal sectors, due to their needle-like shape, they have demonstrated the capability to enter target cells by easy perforation of their membrane, thus transporting their drug cargo inside [60]. Also, CNTs have shown an intrinsic capability to conjugate with several therapeutic molecules, proteins, and genetic material, which can be further improved by appropriate functionalization [60]. All these characteristics make them superior to other nanomaterials in several applications. Nowadays, CNTs have a wide range of possible applications and are increasingly inserted in several nanocomposites, such as thin-film transistors, transparent conducting electrodes, photovoltaics, supercapacitors, printed electronics, e-readers, flexible displays, conductive and/or waterproof paper, catalyst supports, nano-porous filters, and coatings. CNTs are also considered in the I/R optics industry and in the food industry for nanomaterial-improved food packaging [4,55,56]. Also, CNTs find several applications in nanomedicine as biosensors, DNA-based sensors, piezoelectric and gas sensors [61] and are applied for nanomaterials-improved tissue engineering and energy storage medical treatments [55]. CNTs have great potential in nanomedicine for disease diagnosis and delivery of biomolecules, such as proteins, DNA, RNA, immune-active compounds, and lectins, thus being promising new drug delivery systems and excellent carriers for gene therapy [62,63,64]. Additionally, cationic CNTs have demonstrated interesting antibacterial and antifungal activity [55,61]. SWNTs have influenced a significant amount of activity in both research and industry across the world. Furthermore, the increasing application of SWNTs has stimulated considerable investment in ameliorating manufacturing methods and characteriszation and purification techniques to obtain more precise CNTs without defects and impurities, thus reducing their toxicity and improving the development of safer applications. The main recognised drawback of CNTs consists of their scarce solubility in most solvents, which limits their dispersibility in water and their use in nanocomposite manufacture [59]. To address this issue, several surface modifications have been carried out to increase their hydrophilicity and decrease toxicity [59,65,66]. In addition to advanced synthetic methods, highly efficient purification procedures, and post-synthesis chemical modifications, preventive behavioural conducts have already been developed to limit CNTs’ toxic effects [55]. Furthermore, more and incessant studies should be implemented to make their large-scale production safer and to allow a no-risk extensive utilisation. Despite CNTs being considered the most relevant and valuable nanomaterial ever used until now, the knowledge of their nanotoxicology is still limited. Nowadays, CNTs are receiving substantial interest in cancer therapy as theragnostic [67] and photodynamic therapy (PDT) sensitizers [68] and are very promising materials for use in photothermal treatment (PTT) due to their significant absorption in near-infrared (NIR) areas [4]. CNTs are advantageous for cancer diagnosis and imaging because they can convert laser energy into acoustic signals and display strong resonant Raman scattering and photoluminescence within the NIR range [69]. Many authors have reported that CNTs can enter different kinds of cells due to their needle-like structure, which promotes efficient tumour penetration [70]. CNTs can also easily reach the different constituents of the tumour microenvironment (TME), thus irreversibly disturbing the living conditions of tumour cells and allowing the arrival of therapeutic compounds to various intracellular destinations, including nuclei, mitochondria, and cytoplasm, for synergistic anticancer effects [4]. CNTs are evolving in many biomedical devices, including genetically engineered ones, and are applied in imaging and biosensing to treat and diagnose various cancers [71,72]. Due to their exclusive structural characteristics, CNTs demonstrated great hydrophobicity in water, which confers on them intrinsic cytotoxic properties to tumour cells [73]. Functionalization and modification of pristine CNTs with various chemical groups or biomolecules not only have demonstrated to ameliorate their hydrophilicity, solubility, and dispersibility and to reduce toxicity to normal cells but also have shown to be crucial in bettering their effectiveness for widespread and safer use in cancer therapy [74]. Furthermore, several other factors, including their chemical composition, target cells, and environmental reactions, can affect the CNTs’ biological effects [75].

Table S1, in Supplementary Materials, reports some structural properties demonstrated by several modified and/or activated CNTs compared with those of unmodified ones (first row).

In this paper, we have debated the most relevant advancements about the adjuvant role that CNTs could have in cancer diagnosis and therapy when associated with photothermal therapy (PTT), photodynamic therapy (PDT), drug targeting (DT), gene therapy (GT), chemotherapy (CT), and immunotherapy (IT). Numerous sensing strategies utilising CNT-based electrochemical, colorimetric, and plasmonic biosensors, as well as immunosensors for cancer diagnosis, have been discussed in detail, never forgetting the still not fully clarified toxicological aspects that can derive from their extensive use in biomedicine. The unsolved challenges that still hamper the translation of CNT-based material in clinical anticancer practice, including regulatory hurdles, have been discussed, revealing the existence of only a few clinical trials and no clinical use. The main scope of this review was to provide scientists with a huge amount of updated information on the high potential of CNTs if applied in cancer therapy, to push them to focus on the development of advanced synthetic methods and purification workup procedures, thus achieving more perfect CNTs for their safer real-life clinical use.

## 2. Current Synthetic Methods to Obtain CNTs

Table S2 (Supplementary Materials) includes the main conventional methods extensively explained in our previous works [55,56,76]. Other less-used methods to produce CNTs have been extensively described in Alfei et al., 2022 [56] and listed in Alfei et al., 2025 [55,76]. Collectively, all available methods described in Table S2 (Supplementary Materials) use precursor gases such as CH_4_ and C_2_H_2_, which derive from coal and petroleum raw materials, and Fe, Co, and Ni NPs as catalysts. Therefore, the major limitations of CVD processes consist of the use of non-renewable carbon sources and the large emission of waste gases such as toluene [77], thus worsening energy shortages and environmental pollution [78]. Aiming at developing new environmentally friendly, safe, inexpensive methods to produce CNTs, renewable raw materials as precursors to prepare CNTs, such as biomass, seem to be a promising option to the use of non-renewable gases [76]. Table S3 (Supplementary Materials) collects several examples of CNTs prepared using biomass as an eco-sustainable source of carbon [76]. Operating conditions, types of catalysts, and methods, as well as the CNT types obtainable and their physical characteristics, have also been reported in Table S3 [76].

## 3. Carbon Nanotubes as Delivery Systems for Anticancer Drugs

CNTs have been extensively experimented with as nano vectors for the transport and delivery of various agents, including contrast media and drugs. Drug-based, nucleotide-based, and plasmid-based CNT complexes have been developed and have been tested in vitro and in vivo against several types of cancer. Also, being intrinsically cytotoxic mainly for their needle-like structure, which allows their easy penetration of cell membranes, CNTs associated with existing cytotoxic agents in drugs/carrier complexes were assayed to assess possible synergistic effects. Hybrid nanocomposites based on CNTs have demonstrated great potential for realising cancer combination treatments when associated with different currently available techniques, including photothermal therapy (PTT) and sonodynamic therapy (SDT). CNTs are studied for both the early detection of tumorigenic cells and the treatment of enhanced metastatic cancers. Barber et al. reported that MWCNTs loaded with Pt-NPs and polybenzimidazole (PBI) caused cycle arrest in breast cancer stem cells (CSCs), thus diminishing drug resistance and impeding DNA repair [79]. CNTs embedded with Span, PEG, folic acid (FA), and paclitaxel were capable of easily penetrating breast cancer cells, thus inhibiting their development and inducing tumour cell death [80]. Behazadpour et al. reported that the administration of 250 mg/mL of MWCNTs loaded with poly pyrrole to C540 Male Balb/c mice reduced tumour cell viability to under 9% under multi-step ultrasonic irradiation. A total of 8.9% viable cells presenting 75% necrosis and 50% tumour volume reduction (TVR) were observed after 10 days of SDT [81]. A 6.4 times higher accumulation of CREKA peptide in tumour tissues was observed when the peptide was administered to mice embedded in MWCNTs, with xenograft eradication after four cycles of illumination [82]. Also, higher toxicity to tumour cells was observed when PEG-O-CNTs, O-CNTs, and pure CNTs were administered to both tumour cells and female mice under a continuous-wave NIR laser diode (808 nm) for 10 min., with higher TVR in the animal group administered with PEG-O-CNTs [83]. Table 1 schematically summarises the above-mentioned papers where various CNT-based nanomaterials have been used as drug carriers in vitro and in vivo to treat several forms of cancer in association with PTT and SDT. 

### 3.1. Carbon Nanotube-Based Drug Delivery Systems for Target Anticancer Therapy

**Figure 2:**
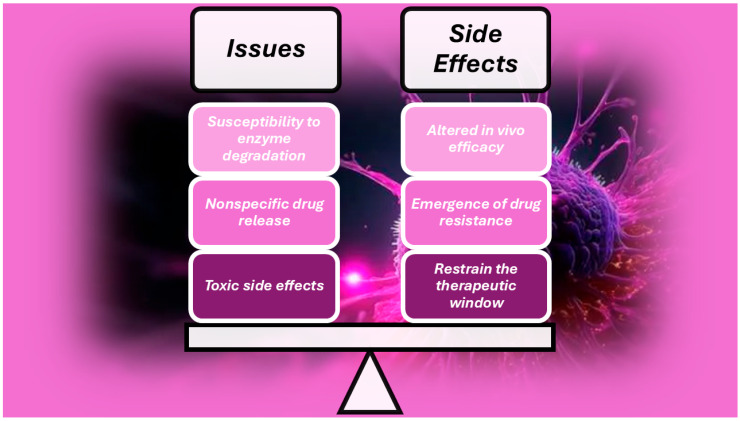
Limitations and associated undesired side effects of chemotherapy for treating tumours.

Cancer diseases are usually treated using chemotherapy associated with surgery and radiation, with good achievements in terms of tumour mass reduction and its expansion limitation, but nonspecific drug release and toxic side effects to normal cells can promote drug resistance and restrain the therapeutic window [85]. Moreover, other limitations are the susceptibility of drugs towards enzyme degradation and denaturation, which can alter their in vivo efficacy (Figure 2).

To limit such issues and improve the therapeutic efficacy of chemotherapeutics while reducing their toxicity towards normal cells and enhancing the patient’s compliance with chemotherapy, new techniques for specific drug targeting have emerged [86,87]. Specifically, nanotechnology provided several nanomaterial-based delivery systems (DSs), which demonstrated high potential to solve most of these issues. Nanomaterial-based DSs can accumulate near tumour sites, thanks to enhanced permeation and retention (EPR) effects, due to more penetrable blood vessels and the missing lymphatic drainage in tumour tissue [88]. When administered in vivo, drugs enveloped in nanomaterials are protected from the degradative environment, while the nanocarriers could be capable of providing drug release in a controlled and protracted mode [89]. Among nanomaterials, CNTs possess unparalleled properties, such as large surface area and high aspect ratio, thus allowing high loading capacity and making them ideal candidates for efficient drug delivery [90]. Also, CNTs showed the capability to realise pH-dependent sustained drug release and enhanced cellular internalisation, as well as demonstrated high stability and the possibility of post-synthesis modification to further enhance their original properties [90]. Table 2 summarises the most relevant functionalizing moieties, which have been used to functionalize/modify pristine CNTs, and the applications of the obtained CNT derivatives in cancer therapy.

**Figure 3:**
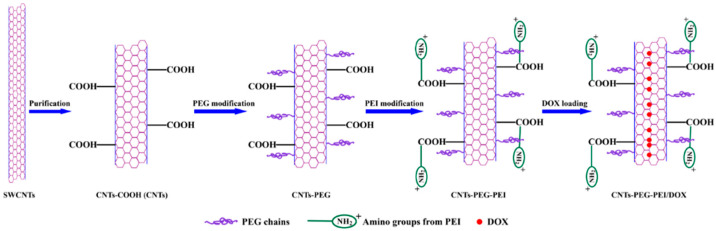
Schematic representation of -COOH modified SWCNT-PEG-PEI nanocarriers and the DOX-loading process [100]. The image has been reproduced by a paper published in diamond open access under the CC BY 4.0 licence (https://creativecommons.org/licenses/by/4.0/, accessed on 24 May 2025), which permits you to copy and redistribute the material in any medium or format for any purpose, even commercially.

The following Figure 3 schematizes the structure of a -COOH polyethylene glycol (PEG) and polyethylene imine (PEI) modified SWCNT-based nanocarrier, developed by Yang et al., and the doxorubicin (DOX)-loading process [100].

**Figure 4:**
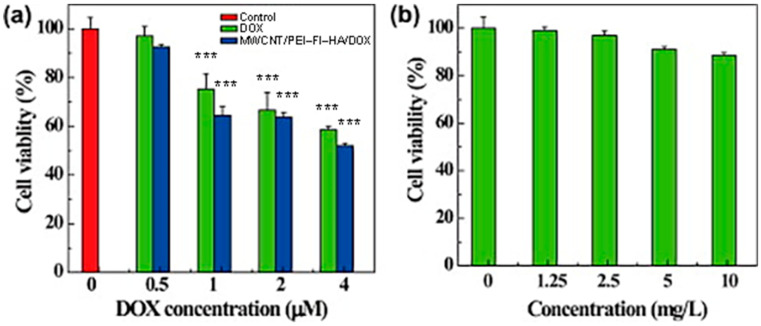
(**a**) MTT viability assay of HeLa cells treated with free DOX and MWCNT/PEI–FI–HA/DOX complexes at the DOX concentrations of 0–4 µM for 24 h (significance versus control condition, *** = *p* < 0.001), and (**b**) DOX-free MWCNT/PEI–FI–HA at corresponding DOX concentrations of the complexes between 1.25 and 10 mg/L. Reprinted with permission from Carbohydrate Research, Copyright 2015, Elsevier [106]. License number 6018200526333 released on 29 April 2025 by Elsevier and Copyright Clearance Centre.

Yang et al. observed that SWCNT@PEG@PEI@COOH DSs displayed higher anticancer effects against MCF-7 cells and better drug delivery under acidic conditions than CNT@COOH and CNT@PEG. Flow cytometry and fluorescence experiments evidenced improved internalisation of SWCNT@PEG@PEI@COOH DSs and enhanced tumour cell death via apoptotic mechanisms due to their high dispersibility and greater affinity towards cancer cells [100]. Similarly, Liu et al. prepared PEG-functionalized SWCNTs loaded with DOX, which demonstrated a loading capacity of ~400% by weight and a controlled release rate of cargo [101]. SWCNTs generally exhibited a higher drug loading capacity than MWCNTs [102], while short SWCNTs and MWCNTs showed higher and faster loading capacity than long CNTs [103]. Furthermore, aromatic peptides showed high binding affinity to SWCNTs due to their interaction with the π electrons of their surface [104]. A foremost advantage of using CNT-based nanocomposites to develop cancer drug DSs (CDDSs) consists of their susceptibility to release drugs specifically in the acidic environment existing in tumour tissues, thus allowing selective passive targeting of the tumour site, with reduction of side effects to normal cells. The pH-sensitive release of anticancer drugs from CNTs CDDSs increased drug residence time in circulation and selectivity and decreased administration frequency, with possible major compliance of cancer-affected patients to therapy, while preserving the optimum drug concentration [105]. In this regard, Cao et al. demonstrated that PEI- and hyaluronic acid (HA)-modified MWCNTs designed for the targeted delivery of DOX to cancer cells overexpressing CD44 receptors showed a drug loading capacity of 72% and a higher release rate in acidic pH (5.8 pH in cancer) than in physiological conditions (pH 7.4 in normal cells) [106]. Therefore, the PEI@MWCNT@HA@DOX nanocomposite showed good biocompatibility in the tested concentration range while exhibiting substantial cytotoxic effects on cancer cells (Figure 4).

Similar results were reported by Gu et al. for SWCNTs modified with benzoic acid via hydrazine bonding (HBA) loaded with DOX when tested on HepG2 cells [107]. The release of cancer cells occurred at a pH of 5.5 in the tumour microenvironment (TME). Additionally, after 60 h of incubation, the SWCNT@HBA@DOX complex demonstrated a higher release of DOX than the SWCNT@DOX composite (73% drug release vs. only 50%). Also, higher cytotoxic effects were observed for SWCNT@HBA@DOX than for the SWCNT@DOX complex due to enhanced cellular internalisation. Since cancer cells require high amounts of folic acid for DNA synthesis and rapid proliferation, they overexpress folate receptors. Conjugation of DDSs with folic acid (FA) is a common approach for targeting cancer cells [108]. Lu et al. functionalized a magnetic nanocomposite composed of MWCNTs and Fe_2_O_3_ nanoparticles (IONPs) with poly(acrylic acid) (PAA) by free radical polymerisation (FRP), and the obtained nanocomposite was further conjugated to FA and loaded with DOX [109]. When utilised on U87 human glioblastoma cells, the nanocomposite exhibited a dual-targeting effect via both magnetic field and ligand-receptor interaction [109]. The nanocomposite demonstrated higher efficiency than DOX alone due to easier hydrogen bonding interactions and π–π stacking while exhibiting enhanced cytotoxic effects [109]. The higher effectiveness of DOX when delivered by the CNT-based DDS also derives from its efficient internalisation inside the cells and transport to the nucleus, which permits the intracellular release of DOX [109]. The following Table 3 and Table 4 collect various reported experiments that used CNTs for the target delivery of different anticancer drugs and genetic material. Specifically, Table 3 summarises several in vitro and in vivo relevant experiments on the delivery of antitumour drugs and/or nucleic acids using modified CNT-based nanocarriers, while Table 4 collects results about the cytotoxicity to different tumours and biocompatibility results concerning several modified CNT-based anticancer DDSs.

#### 3.1.1. Carbon Nanotubes-Based Drug Delivery Systems for the Tumour Microenvironment (TME) and Site-Specific Target Cancer Therapy

**Figure 5:**
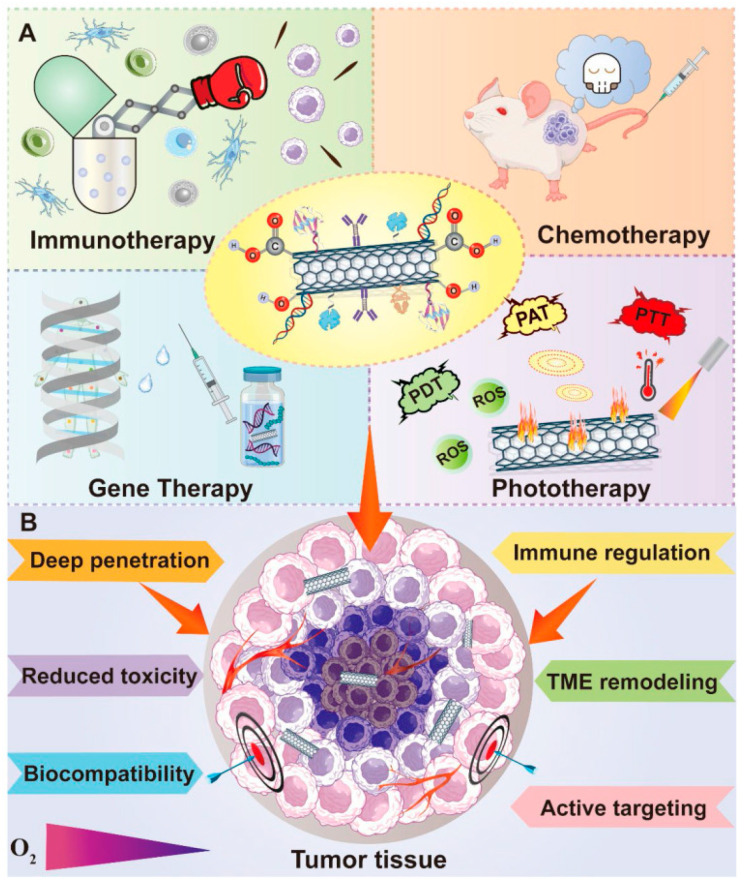
(**A**) Anticancer therapeutic strategies, which can take great advantages if associated with functionalized CNTs. (**B**). Advantages deriving by the application of functionalized CNTs in different therapeutic approaches. PAT = photo-acoustic tomography; PTT = photothermal therapy; PDT = photodynamic therapy; ROS = reactive oxygen species; TME = tumour microenvironment. The image has been reproduced from an open access article distributed under the terms of the Creative Commons Attribution License CC BY 4.0 licence (https://creativecommons.org/licenses/by/4.0/, accessed on 26 May 2025). See also http://ivyspring.com/terms for full terms and conditions, accessed on 25 May 2025 [149].

To effectively manage and precisely remove malignant tumours, a true understanding of cancer features and the interactions between tumour cells and their surrounding microenvironment is necessary. As extensively demonstrated in the previous sections, many anticancer strategies focus on the target delivery of chemotherapeutics to tumour cells to enhance their selectivity and anticancer efficiency while reducing toxic effects to normal cells. The emergence of cancer nanomedicine has allowed huge advancements in the treatment of cancer and in targeted cancer therapy, and several types of nanomaterial-based drug delivery systems have been developed for anticancer target delivery. Among nanomaterials, carbon nanomaterials, including CNTs, and mainly functionalized CNTs, have been demonstrated to be versatile multifunctional platforms, very promising for innovative cancer treatment. In this context, the following Figure 5 shows the anticancer therapeutic strategies, which can take great advantage if associated with CNTs (Figure 5A). Particularly, by using functionalized CNT-based nanocomposites in immunotherapy (IT), chemotherapy (CT), gene therapy (GT), and phototherapy (PT), significantly enhanced anticancer effects, reduced toxicity, and improved biocompatibility can be achieved. These improvements can derive from improved deep penetration, immune regulation, TME remodelling, and active targeting (Figure 5B).

As schematized in Figure 5, CNTs have been successfully experimented on in association with many treatment modalities like chemotherapy, gene therapy, phototherapy, and immunotherapy, and they have resulted in efficient tools to develop CNT-based nanocarriers capable of delivering various anticancer agents to the intracellular sites of interest in cancer cells, such as the nucleus, mitochondria, cytoplasm, and other organelles, thus realising direct tumoricidal effects [8,150]. A promising current approach in cancer therapy regards targeting the components present in the tumour microenvironment (TME) where tumour cells live, thus detrimentally impacting cell survival by impairing their living environment [151,152]. TME is a very complicated system stuffed with various types of cells and full of extracellular matrix (ECM), characterised by altered vasculature, higher acidity and interstitial pressure, abundant glutathione (GSH) levels, poor blood perfusion, abnormal metabolism, and hypoxia [149]. Collectively, TME is essential for tumour establishment and progression and represents the obstacle that limits the efficacy of numerous cancer treatment approaches [153]. Furthermore, despite the immunosuppressive properties of TME promoting the tumour cells’ resistance to immunotherapy and despite it producing a lot of impediments to effective cancer treatment, TME can also be exploited as a therapeutic target, where abnormalities can be exploited for the development of new selective anticancer strategies. In this context, many innovative anticancer approaches, such as ECM modulation, angiogenesis and cancer stem cells (CSCs) inhibition, immunoregulation, and TME-responsive controlled drug delivery, aimed at remodelling and impairing TME, have been widely studied (Figure 5). CSCs’ inhibition strategy has been experimented with by Faraj et al. in an attempt to address the depressing failures of many currently adopted treatments in the cure of breast cancer. They designed multimodal nanoplatforms based on SWCNTs to achieve non-invasive imaging and specific targeting towards breast CSCs [154]. To this end, SWCNTs were modified with PEG, and since CD44 is a surface marker of breast CSCs, PEGylated SWCNTs were conjugated with CD44 antibodies, thus realising active targetability towards breast CSCs. Magnetic resonance imaging (MRI), single photon emission computed tomography (SPECT), and NIR fluorescence techniques evidenced an enhanced selective tumour-targeting phenomenon in MDA-MB-231 tumour-bearing mice. Moreover, it was observed that PEG@SWCNTs@anti CD44 distributed specifically in the tumour sites where CD44 receptors are abundant, thus further confirming an elevated targetability to CSCs. As previously reported, the immunosuppressive power of TME often prevents the immune system from effective tumour eradication. Aiming at addressing this problem, Hassan et al. employed MWCNTs as DDSs for the co-delivering of immunoadjuvants such as CpG, anti-CD40 Ig, and OVA antigen for enhanced immunotherapy. The covalent conjunction of OVA and CpG remarkably elevated the responses of OVA-specific T cells both in vitro and in C57BL/6 mice, while the subsequent loading of anti-CD40 Ig amplified the antitumour immune reactions. Using MWCNTs, the co-loading ability of the three ingredients was improved, translating into a significant inhibition of tumour growth and metastasis in the OVA-expressing B16F10 melanoma model. Also, Tang et al. engineered a co-delivery platform based on PEI-functionalized MWCNTs to inhibit angiogenesis in lung cancer TME for its treatment [147]. Integrin ανβ3 is strictly correlated with angiogenesis, thus representing a therapeutic target for anticancer therapy. RGD peptides are capable of binding to integrin ανβ3, thus being a targeting ligand for anticancer drug delivery [155]. Fu and co-workers connected the iRGD peptide and candesartan, an angiotensin receptor blocker, to PEI-modified MWCNTs and assembled the achieved nanocomposite with plasmid angiotensin II type 2 receptor (pAT2) through electrostatic interaction. A multifunctional DDS was achieved, which demonstrated the capability of successfully delivering candesartan and pAT2 into tumour cells, thus significantly inhibiting tumour growth and neovascularization in the A549 lung cancer model. Table 5 reports several examples of CNT-based DDSs that demonstrated the capability to deliver the transported material to specific intracellular sites and TME constituents.

#### 3.1.2. Carbon Nanotubes-Based Drug Delivery Systems for the Tumour Microenvironment (TME)-Responsive Release of Chemotherapeutics

The enhanced permeability and retention (EPR) effect is a concept [173,174] establishing that small molecules, typically liposomes, nanoparticles, and macromolecular drugs, tend to accumulate in tumour tissue much more than they do in normal tissues [175,176]. The conventional drug delivery systems (DDSs) work mainly by exploiting the EPR effect and receptor-mediated endocytosis. Unfortunately, they have habitually had to overcome many obstacles due to the biological intricacy of the tumour microenvironment (TME), where cancer cells reside [177]. Furthermore, the acidic and hypoxic characteristics of TME and the high concentrations of glutathione (GSH) and H_2_O_2_ allow us to conceive TME-responsive drug release tactics. Activated by several stimuli deriving from the conditions existing in the TME, the drugs transported by these appositely engineered DDSs can be released in a controlled or sustained mode, thus realising various therapeutic effects and specifically penetrating deeply into the tumour tissue. Moreover, the reaction occurring between drug-loaded DDSs and these sensitive factors can lessen tumour hypoxia and acidity, thus creating an environment hostile to tumour cells and more suitable for better therapeutic results. In this context, Yang et al. ideated oxidised MWCNTs gifted with a large inner diameter, which allowed them to entrap cisplatin inside and to load doxorubicin (DOX) on the surface [178]. To hamper the early release of cisplatin, polyethylene glycol (PEG) and folic acid (FA) were also employed. With this approach, the carried cargos were endowed with a pH-sensitive release profile and were released under pH = 6.5, which is just the weak acidic condition existing in TME. When tested on MCF-7 breast cancer cells, the nanocomposite demonstrated a more marked cytotoxicity in the pH = 6.5 condition than in pH = 7.4, confirming its best capacity to specifically kill tumour cells in acidic TME. Zhang et al. engineered flexible cotton cellulose-incorporated MWCNT working as pressure sensor which exhibited a sensitivity of about 0.0197 kPa^−1^, a response time of about 20 ms, a recovery time of about 20 ms, and a wide workable pressure range from 0 to 20 kPa [179]. The proposed pressure sensors are prospective for various applications including cancer diagnosis, and treatment [179]. A uniform MnO_2_ sheet merged with Ce6 photosensitizer was modified with MWCNTs, achieving a nanosystem for enhanced phototherapy. MnO_2_ was used because it was capable of rapidly depleting GSH through the redox reaction of Mn^4+^ into Mn^2+^ ions and of decomposing H_2_O_2_ present in the TME to give ^1^O_2_. By this approach, the photothermal effect engendered by MWCNTs was encouraged, tumour hypoxia was reduced, and Ce6-mediated PDT was eased. ROS-mediated cell death increased via chemo-dynamic therapy, and Ce6 release was sped up. Overall, this multifaceted MWCNT-based platform could represent a promising strategy to achieve synergistic cancer diagnosis and therapy. Also, Qin et al. developed a CNT-based nanoplatform encapsulated into a certain thermos-/pH-sensitive nanogel, thus obtaining a DDS capable of near-infrared (NIR)-triggered, TME-responsive drug release [180]. Upon loading with DOX, this nanosystem showed a quicker release rate of DOX at 40 °C than at 25 °C and at pH = 5.0 than at pH = 7.4, which indicated combinational outcomes due to CNTs-mediated photothermal effects under NIR irradiation and TME-responsive drug release. The following Table 6 collects the above-mentioned case studies and several other ones.

## 4. Carbon Nanotubes Application in Anticancer Phototherapy

### 4.1. Anticancer Photothermal Therapy

As an unconventional anticancer therapeutic technique, photothermal therapy (PTT) is part of a family of minimally invasive strategies that are based on the use of photosensitizers. Specifically, PTT kills cancer cells thermally by creating local heat using an optical absorption mediator, that is, a photosensitizer, capable of absorbing electromagnetic energy (EME) and transforming it into heat [181]. By generating a condition of hyperthermia, tumour eradication is significantly improved by boosting immune activation and causing immunity towards metastatic cancer cells for a long time [182]. Unfortunately, conventional photosensitizers are affected by several drawbacks, including undesired adverse effects on skin, limited targeting of cancer cells, and scarce therapeutic effects in hypoxic TME. In this regard, CNTs represent excellent next-generation photosensitizer agents for a more effective PTT. They possess better photophysical properties and the ability to target cancer cells and accumulate in the tumour site. They are gifted with a broad electromagnetic absorbance spectrum and the capability to convert near-infrared I and II windows, which match the optical transmission window of biological tissues [183]. Moreover, the use of CNT-based tumour-targeting conjugates, in combination with PTT treatment, can result in more precise and efficient tumour elimination. Using well-dispersible PEG-wrapped CNTs containing 80% (*w*/*w*) PEG on the CNTs surface, Sobhani et al. demonstrated the effectiveness of PTT treatments against HeLa and HepG2 cells [83]. Moreover, when PEG@CNTs were evaluated against melanoma using a tumour-bearing mice model exposed to a continuous-wave near-infrared laser diode for 10 min, a more significant reduction in tumour size was observed in mice receiving PEG@CNTs associated with laser irradiation than in mice receiving only the laser radiation. Similarly, Zhu et al., avoiding the use of surfactants, prepared biocompatible, normal cell-friendly, and nontoxic hybrid complexes, encompassing MWCNTs and gold nanostars (MWCNTs@Au@NSs) [184]. MWCNTs@Au@NSs were experimented on in association with PTT on B16F10 mouse melanoma cells, observing that under 808 nm radiation, a photothermal effect 12.4% and 2.4 times higher than NSs and Au@NSs, respectively, was produced, which caused enhanced cancer cell death. Also, by merging PTT with immune stimulation by means of annexin A5 (ANXA5)-conjugated SWCNTs and anti-CTLA-4 checkpoint inhibitors, McKernan et al. tried to treat metastatic breast cancer cells [185]. The designed combined anticancer therapy caused a significant increase in the survival rate of mice and in the number of CD4^+^ helpers and CD8^+^ cytotoxic T cells, while SWCNTs had no toxic effects during the experiment. Moreover, even if the targeted and controlled release of genes remains a major challenge, Zhao et al. showed the synergistic effect of SWCNTs/MWCNT-based PTT and gene therapy in exerting antitumour activity [156]. SWCNTs and MWCNTs were coated with peptide lipid and sucrose laurate to form a bifunctional DDS with improved photothermal effects and temperature sensitivity, which was loaded with siRNA. The complex silenced the survivin expression, thus effectively repressing tumour growth, while exhibiting photothermal effects under NIR exposure. Peptide lipid and sucrose laurate facilitated the phase transition of lipids, thus enabling the systemic delivery of siRNA to the tumour site. SWCNTs produced by the CoMoCAT® method possess high nanotube chirality, display an absorption band at 980 nm, and photothermal activity, which was widely used for selective photo-tissue interaction [185,186]. In this regard, with the aim of targeting folate receptors on the surface of cancer cells, Zhou et al. coupled CoMoCAT®-SWCNTs with folic acid (FA) [187]. In vitro and in vivo experiments demonstrated that the conjugate significantly reduced the photothermal destruction of normal cells while significantly enhancing the photothermal death of tumour cells. Fibrin is the final product of the coagulation response, which is highly concentrated at the vascular injury site [188]. Fibrin existing in the tumour vessels can serve as a therapeutic target for drug delivery due to its easy accessibility, ubiquitous presence, and high expression. On these considerations, Zhang et al. engineered fibrin-targeting CREKA peptide-conjugated PEG@MWCNT nanocomposites for the PTT of cancer cells [82]. Exposure of a tumour-bearing mouse model to MWCNT@PEG significantly increased the temperature of the tumour site after 24 h of NIR radiation. It was shown that, upon illumination, IR783-labelled MWCNT@PEG accumulation in the tumour site was 6.4 times higher than in the control group [82]. MWCNT@PEG completely suppressed tumour xenografts after four illumination cycles. Overall, MWCNT@PEG demonstrated significant tumour targeting and photothermal therapeutic effectiveness. Suo et al. proposed MWCNTs coupled to a Pgp-specific antibody (Pab) for photothermal P-Glycoprotein (Pgp)-mediated extirpation of MDR ovarian cancer cells [82]. Results demonstrated a remarkably improved internalisation of the Pab–MWCNTs in 3T3-MDR1 than in 3T3 cells at different time periods. Under NIR irradiation, Pab–MWCNTs demonstrated higher dose-dependent specific photokilling in 3T3-MDR1 than in 3T3 cells [82]. Due to the higher temperatures of cancer cells reachable by the plasmon phenomenon, differently modified MWCNTs demonstrated higher cytotoxicity to cancer cells. The highest temperatures for MWCNT-COO, MWCNT-COOPt, and MWCNT-Pt were 43.4 °C, 45.8 °C, and 46.2 °C, respectively, while those of MWCNT-COO, MWCNT-COOAu, and MWCNT-Au were 44.1 °C, 46 °C, and 46.9 °C, respectively [189,190].

### 4.2. Anticancer Photodynamic Therapy

Another unconventional anticancer therapeutic technique, included in the family of non-invasive strategies against cancer and based on the use of photosensitizers, is the low-toxic photodynamic treatment (PDT). PDT utilises a combination of light, chemical photosensitizers, and molecular oxygen to induce cell death [86]. Briefly, upon the topical or systemic administration of a photosensitizer to tumour cells, it can be activated by light at a specific wavelength (NIR) [86]. An energy transfer cascade occurs that induces ROS overproduction in the presence of oxygen, resulting in selective cytotoxicity against cancerous cells [191]. Racheal et al. engineered zinc phthalocyanine@spermine@SWCNTs and compared them to mono carboxy phenoxy phthalocyanine, not containing zinc, and zinc mono carboxy phenoxy phthalocyanine-conjugated spermine deprived of the SWCNTs. Photophysical properties and PDT efficiency towards MCF-7 breast cancer cell lines were assessed [192]. Results demonstrated that both ZnMCPPc-spermine and ZnMCPPc-spermine-SWCNT possessed photophysical characteristics superior to those of zinc-free phthalocyanine, with >50% improvements in triplet and singlet oxygen quantum yields. In vitro cytotoxicity experiments carried out using MCF-7 cancer cells evidenced that the PDT carried out using ZnMCPPc-spermine and ZnMCPPc-spermine-SWCNT caused 97% and 95% cell viability reduction, respectively, at 40 mM. Also, the same Racheal, with colleague Nyokong, assessed the PDT outcomes of a Zn-phthalocyanine modified with SWCNTs in association with ascorbic acid against MCF-7 tumour cells [193]. ZnMCPPc, ZnMCPPc@AA, ZnMCPPc@SWCNT, and ZnMCPPc@AA@SWCNT were evaluated for their photophysical properties (PPPs) and PDT performance (PDTP). ZnMCPPc@SWCNTs demonstrated enhanced PPPs, prolonged lifetimes, and improved singlet oxygen quantum yields with respect to ZnMCPPc alone. ZnMCPPc@SWCNT showed excellent PDTP against MCF-7 cells, leading to a 77% cell viability reduction. Sundaram et al. studied the PDTP of SWCNTs-modified hyaluronic acid (HA) and chlorin e6 (Ce6) nanocomposites (NCs) on colon cancer cells [194]. The HA coating significantly enhanced the dispersibility of NCs. The as-synthesised SWCNT@HA@Ce6 NCs proved to have higher anticancer effects on Caco-2 cells than free Ce6. Also, they showed improved capacity to deliver photosensitizers, as well as stronger apoptotic activity in colon tumour cells, due to a high surface area and strong binding capacity [195]. Nano-bio-composites associated with PDT (both 5 and 10 J/cm^2^ laser irradiated) triggered both early- and late-stage apoptotic death to a greater extent in cancer cells than in control ones. Specifically, exposure to 10 J/cm^2^ (41.9, 6.65) caused more early-stage apoptosis than 5 J/cm^2^ (36, 6.4). Similarly, surviving cells were in lower percentages when exposed to 10 J/cm^2^ (53.4, 6.8) than to 5 J/cm^2^ (58.27, 5.9). Moreover, SWCNT@HA@Ce6 NCs under 10 J/cm^2^ laser irradiation showed higher apoptotic effects than those displayed by free Ce6 and empty SWCNTs. It was reported by Shi et al. that HA-conjugated CNTs NCs were endowed with tumour-targeting capability and improved solubility [196]. CNTs were modified with hematoporphyrin monomethyl ether (HMME) PDT agent, achieving CNT@HA@HMME NCs, which were capable of combining local selective PDT with exterior near-infrared PTT, thus exerting improved therapeutic efficacy and reduced toxicity to normal cells in the treatment of cancer by a synergistic effect [196]. Overall, HMME@HA@CNTs could perform both PDT and PTT treatment simultaneously in future enhanced tumour therapy.

### 4.3. Enhanced Anticancer Phototherapies by CNTs-Improved Drug Delivery

Light-induced hyperthermia in PTT and PDT frequently needs high-power intensity, which may damage nontarget normal cells [197]. To address this issue, Daquan Wang designed a nanoplatform (NP) by coating cut MWCNTs (C-MWNTs) with poly-N-vinyl pyrrole (PVPy) and binding folic acid (FA)-polyethylene glycol (PEG)-SH with thiolene, achieving MWCNT@PVPy@S@PEG@FA nanocomposites (NCs). NCs possessed a high drug-carrier ratio, pH-sensitive release capability of doxorubicin (DOX), and broad-spectrum anticancer effects. These features depended mainly on their exceptional photothermal conversion efficiency and capacity for high loading of DOX and its target delivery due to the presence of the PVPy shell [198]. Similarly, Oh et al. used DOX-loaded SWCNTs to exert NIR cancer PTT chemotherapy [162]. Conversely, FA and methotrexate (MTX) were linked to the surface of -COOH-modified MWCNTs (COOH-MWCNT) by using ethylenediamine (ED) as a coupling agent, achieving MWCNT@ED@FA and MWCNT@ED@MTX NCs, demonstrating the NIR- or IR-laser-promoted highest rate of MCF-7 cancer cell death. Cell death was also high at low doses of MTX when the laser light and MTX in MWCNT@ED@MTX or the laser light and MWCNT@ED@FA worked together [199]. Thermal tumour suppression triggered by CNTs was exploited by Znang et al. to treat cancer cells by chemotherapy and PTT using an association of MWCNTs, gemcitabine, and lentinan, an anticancer drug and an immune stimulator, respectively [200]. Under 808 nm laser radiation, the proposed NC was more efficient in penetrating and suppressing cancer cells and less toxic than each system ingredient when used alone. Also, CNT-based NCs give the possibility to combine therapeutic and diagnostic activities. CNTs can be associated with other nanoparticles of different origins to produce synergistic therapeutic and diagnostic effects [201]. Karimi et al. investigated the cytotoxic effects of MWCNT@MTX and MWCNT@MTX@PEI@FA under 808 nm laser radiation, without observing noticeable differences [202]. DOX or CpG entrapped onto MWCNTs significantly increased the water dispersibility of MWCNTs. When melanoma-bearing mice were exposed to such modified MWCNTs under 808 nm NIR laser radiation at a dose of 1 W/cm^2^ for 5 min, suppression of tumour growth and an improved number of lymph-draining CD4^+^ and CD8^+^ T cells were observed in the spleen, with increased antitumour efficacy [172]. PTT using targeted SWCNTs associated with immune system activation using a checkpoint inhibitor was proposed as an innovative treatment for the management of metastatic breast cancer. Selective NIR photothermal ablation of the primary orthotope at an energy and power level of 175 J/cm^2^ and 1 W/cm^2^, respectively, enhanced the anti-cytotoxic T lymphocyte-dependent abscopal response, translating into an increase in survival rate (55%) 100 days after tumour vaccination. The Annexin A5-functionalized SWCNTs, SWCNTs@ANXA5 NC, were administered systemically before PTT [185]. Biocompatible 3D CNT@MXene@DOX microspheres were engineered, displaying unparalleled photothermal effects and photothermal stability under 650 and 808 nm NIR laser radiation. As-manufactured microspheres were endowed with a maximum drug loading capacity of 85.6% for DOX. Also, 3D CNT@MXene microspheres effectively created singlet oxygen due to the TiO_2_ photosensitisers present on their surface. In vitro experiments showed that 3D CNT@MXene@DOX successfully inhibited HeLa cancer cell proliferation [203].

### 4.4. Application of CNTs for Enhanced Anticancer Combined PTT and PDT

**Figure 6:**
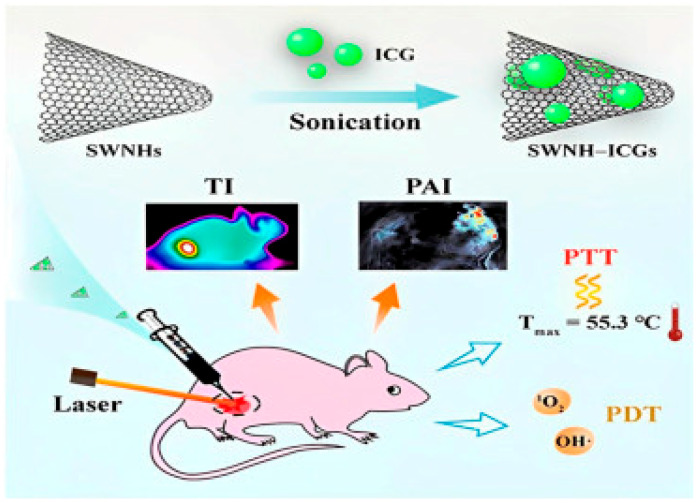
Thermal/photoacoustic imaging-guided PTT and PDT synergistic therapy nanoplatform developed by Gao et al. This image is published [204], and it has been reproduced under licence number 6027091475870 provided by John Wiley and Sons and Copyright Clearance Centre on 13 May 2025 (available online at Rightslink^®^ by Copyright Clearance Center, accessed on 13 May 2025).

Single-walled carbon nano horns (SWCNHs) are a particular type of CNTs characterised by high photothermal conversion efficiency (PCE), which were studied to develop nanoplatforms suitable for different phototherapies, thus enabling the engineering of nanocomposites for combined PTT and PDT. In all relevant articles where PDT and PTT were experimented on in combination, an increase in the pace at which tumours were reduced in animal models was evidenced. Additionally, to further improve this impact, such a combination can also be associated with another treatment, such as chemotherapy. In this context, Gao and co-authors developed an SWCNH@indocyanine green (ICG) theragnostic nano system, which was experimented with for diagnosis and combined PTT and PDT in breast tumour cells at low laser power, observing enhanced synergistic antitumour effects. Particularly, SWCNH@ICG induced ROS overproduction and hyperthermia effects under light radiation. The mechanism that led to killing the tumour and slowing its growth is shown in Figure 6 [204].

The same authors created a similar platform, this time containing hypericin (Hyps). Upon laser radiation at 590 and 808 nm, this nanocomposite functioned as a dual agent for simultaneous PDT and PTT against 4T1 cells implanted subcutaneously in mice as tumour models. Optimum outcomes in terms of anticancer impact by induced hyperthermia and ROS overproduction were observed [205]. Yang et al. immobilised Ce6 and Gd^3+^ on the surface of polymer-coated SWCNHs. Authors investigated its effects when experimented with in combined PDT and PTT against tumours associated with the immune system’s response [206]. High tumour targeting, enhanced tumour penetration efficiency, and immune adjuvant effects were evidenced [206]. The possible synergistic effects of PDT and PTT were investigated by Yin et al., using a CNT-based nanocomposite comprising Ce6 and MnO_2_ to prevent tumour hypoxia under radiation treatments at both 660 and 808 nm to activate both therapies. Despite tumour growth being inhibited also by each therapy used independently, the PS, PA, MnO_2_, and Ce6-MnO_2_@CNTs (CMCs) compounds produced the best outcomes when applied in simultaneous PDT and PTT by synergistic effects [207]. Zhang et al., in addition to combining PDT with PTT, associated PDT and PTT with CT, developing a nanoplatform including both SWCNTs and carbon quantum dots (CQDs). To provide outstanding selectivity towards tumour cells, the water solubility of the nanoplatform was improved using PEG, and it was gifted with magnetic characteristics to allow IMR using Fe_3_O_4_ NPs, and DOX was loaded, thus engineering the SWCNTs@PEGFe_3_O_4_@CQDs@DOX@Apt nanocomposite. Despite all groups who receive radiation outperforming those who receive only CT treatment, when the combination of PTT, PDT, and CT was used, the tumour was suppressed, thus establishing its superiority for treating tumours [208]. Also, Marangon et al. engineered a nanocomposite for treating SKOV3 ovarian cancer cells, using a combination of PDT and PTT. It was based on MWCNTs and m-tetra hydroxyphenyl chlorin (m-THPC) as a photosensitizer. The photothermal and photodynamic cytotoxic effects of m-THPC@MWCNTs complexes were investigated at the cellular level by several methods, including viability tests, analysis of apoptosis-related proteins, genomic analysis of 84 genes involved in OS, etc. The combination of PDT and PTT therapy resulted in cancer cell death by the activation of various signalling pathways and by suppressing the cell’s defence against OS [209]. Then, as previously reported, a new PDT agent, namely HMME, was adsorbed onto HA-modified CNTs (HA@CNTs), thus creating HMME@HA@CNTs nanocomposite, endowed with high aqueous solubility, neutral pH, and tumour-targeting activity. When this new nanocomposite was used in combination with PTT and PDT to treat tumours in vivo and in vitro, improved anticancer efficacy and low toxicity to normal organs were observed due to the capacity of HMME@HA@CNTs NPs to combine local selective PDT with exterior NIR PTT, thus achieving a synergistic impact [196]. The following Table 7 and Table 8 collect some relevant case studies reporting on the application of CNTs in anticancer PTT and PDT, while Table 9 summarises reports on enhanced anticancer phototherapy by CNT-based drug delivery. Lastly, Table 10 collects case studies on the application of CNTs to realise nanoplatforms to perform a combination of PTT, PDT, and CT.

## 5. Carbon Nanotubes Application in Anticancer Gene Therapy

Gene therapy (GT) is a novel engineered therapeutic approach that aims at using genes to cure several diseases, including cancer, by replacing the defective genome of diseased cells with a healthy one [216]. In GT, the opportunistically selected healthy genes are inserted into the selected cells, including cancerous ones, by transfection to repair defects in their genome or compensate for the cells’ deficiencies due to uncontrolled mutations that inexplicably occurred [216]. GT and its synergistic combination with CT are gaining strong interest in cancer treatment. Unfortunately, insufficient endosomal escape of genes/nanocarrier complexes, due to an inadequate buffer capacity of the carrier, causes early lysosomal degradation [86,216]. This event causes poor transfection capability and strongly limits the therapeutic applicability in vivo of this approach. The use of CNT-based carriers, opportunely modified to enhance their buffer capacity, can promote endosomal effects and survival of the gene complex, thus significantly ameliorating the transfection efficiency. The use of suicide genes is among the most efficacious approaches to realising efficient anticancer gene therapy. It consists of using therapeutic transgenes to express toxic products from a toxic gene or to convert a nontoxic prodrug into a toxic one, or both, to fight the effects of cancer disease. This strategy was essayed to treat several cancers, such as breast [217], liver, colon [218], prostate [219], glioma [220], and lung cancer [221], also when cancer cells have acquired chemo-resistance [222]. Additionally, this approach has been demonstrated to enhance the efficacy of radiation therapy [223]. Furthermore, combination therapy is usually more efficient than monotherapy due to its capability to elude the cell cycle arrest caused by chemical drugs. Several studies were developed based on the above-mentioned considerations. Cao et al. engineered a novel pH-responsive SWCNTs carrier functionalized with PEI-betaine (PB) (SPB), improved with BR2 peptide and loaded with DOX and survivin siRNA (SPB@BR2@DOX@sur/siRNA), for the co-delivery of an anticancer drug and a silencing gene and realising efficient anticancer gene therapy [144]. When administered to both HeLa and A549 cancer cells and 293T normal cells, the nanocomposite was selectively internalised into cancer cells, while it did not enter normal cells [144]. The nanocomposite caused less survivin expression and a higher apoptotic index than Lipofectamine 2000 due to the release of siRNA/DOX into the A549 cell cytoplasm and nuclei without lysosomal degradation. In comparison to SPB@BR2@siRNA or SPB@BR2@DOX separate treatments, that in association using SPB@BR2@DOX@sur/siRNA demonstrated synergistic effects, causing significant reduction in the volume of tumour both in A549 cells and in nude mice [144]. Based on the capability of the iC9 suicide gene to induce apoptotic death in MCF-7 human breast cancer cells, Dargah et al. used pyridine-modified MWCNTs (pyr@MWCNTs) as carriers to transfect iC9, achieving the pyr@MWCNTs@iC9 complex [224]. Upon its administration, MCF-7 cells were exterminated, and when associated with chemotherapy, they evaded cell cycle arrest. Also, gene regulation and anticancer therapy were attempted by Zhang et al., who experimented with the chitosan-modified fluorescent carbon nanoparticle (FCN)-based siRNA conjugate (Ch@FCN@siRNA) [225]. The core-shell nanocomposite comprised a core made of Ch@FCN and a shell of siRNA. siRNA down-regulated the key regulator of mitosis, namely polo-like kinase-1 (PlK1) expression. Notably, only a concentration of FCN 30 times lower than that of AuNPs was sufficient to transfect the same amount of siRNA. The nanocomposite, in vitro treatment of A375 and MCF-7 tumour cells, was better performing than the commercial Lipofectamine 2000, inducing 31.9% and 20.33% apoptosis, respectively. Also, its intravenous administration to mice bearing the A375 tumour cells reduced tumour volume by 11-fold compared with control groups. As reported in previous sections, Guo et al. used MWCNTs as efficient siRNA vectors. Precisely, the authors modified MWCNTs with NH_3_ groups, which were cationic at physiological pH, obtaining positively charged MWCNTs-NH_4_^+^ tubes, which were used for siRNA delivery against PLK-1 cancer cells in mice [125]. Upon the administration of siRNS@MWCNTs-NH4^+^ complex, lung cancer xenografts were eradicated [125]. Another study by Anderson et al. used SWCNTs for the targeted delivery of siRNA to pancreatic cancer cells for anticancer gene therapy [226]. In this contribution, the prepared SWCNTs@siRNA complex was tested in vitro on pancreatic cancer cells, observing high siRNA transfection efficiency, successful internalisation of the nano complex in cancer cells, and low toxicity versus normal cells [226]. The release of siRNA from the nano complex resulted in the downregulation of the target oncogene [226].

## 6. Carbon Nanotubes Application in Anticancer Immunotherapy

Immunotherapy is a therapeutic approach that is designed to improve the patient’s capability to fight several types of diseases, including cancer, by either the modification or amplification of the immune system by exploiting antigenic targets [227]. This therapeutic strategy has proven to be effective in enhancing therapeutic effects against chronic infections and cancer. Another anticancer strategy consists of blocking regulatory systems that may interfere with the immunotherapeutic effects. In the body, dendritic cells (DCs) represent a crucial connection between innate and adaptive immunity. They are the most potent specialised antigen-presenting cells (APCs), playing a pivotal role in anti-infection and antitumour responses. Unfortunately, cancer cells can overpower both the immune system and the functionality of DCs, limiting the efficacy of DC-based antitumour immunotherapy. Hence, it could be of paramount help in improving the antitumour immune response by controlling DCs’ functionality and disabling immune tolerance. Also, cytotoxic T lymphocytes (CTLs), also known as CD8^+^ T and CD4^+^ T cells, are crucial cells of the adaptive immune system. They play a determinant role in the defence against pathogens such as viruses, bacteria, and tumours [228]. An inadequate infiltration of CD8^+^ T cells in the immunosuppressive TME results in a decreased antitumour response. When CD^+^ T cells are even absent, the body will lack antitumour immune function. Conversely, the over-presence of CD8^+^ T cells can trigger excessive immune responses, leading to immune-mediated tissue damage or pathological reactions [229]. Thus, enhancing CD8^+^ T cell infiltration in TME, as well as promoting their correct functional activity, are pivotal strategies in tumour treatment. Carbon-based nanoparticles, including CNTs, could be supreme platforms for tumour detection and immunotherapy. Several research studies indicate that polymer-modified CNTs can treat tumours by acting as immune adjuvants to promote the maturation of dendritic cells (DCs), the CD8^+^ T cell infiltration in TME, and the release of antitumour factors [230]. Furthermore, MWCNTs conjugated with peptides can promote cytokine secretion, stimulating T cell differentiation and proliferation [231].

**Figure 7:**
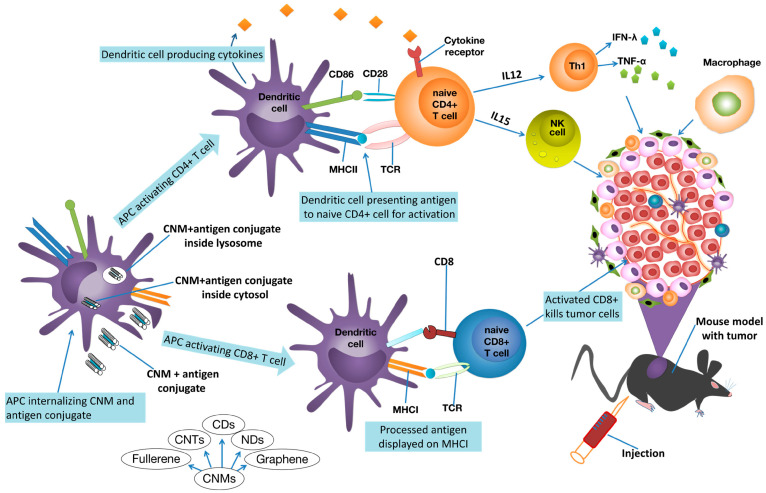
Schematic representation of the immunomodulatory effects of CNMs in cancer therapy [236]. The image has been reproduced by an open access article [236]. Reproduction is licensed under a Creative Commons Attribution 4.0 International License, which permits use, sharing, adaptation, distribution, and reproduction in any medium or format. To view a copy of this licence, visit http://creativecommons.org/licenses/by/4.0/ (accessed on 6 July 2025). The Creative Commons Public Domain Dedication waiver (http://creativecommons.org/publicdomain/zero/1.0/, accessed on 6 July 2025) applies to the data made available in this article, unless otherwise stated in a credit line to the data. APCs = antigen-presenting cells; DCs = dendritic cells; CNM = carbon nanomaterial; CNTs = carbon nanotubes; CDs = carbon dots; NDs = nanodots; MHC-II = histocompatibility complex II; CD4^+^, CD8^+^ = helper T cells; MHC-I = histocompatibility complex I; CTL = cytotoxic T lymphocyte; NK = natural killer.

Carbon-based nanomaterials (CNMs) and CNTs can augment antitumour immunity of patients via multiple and several mechanisms of immune system modulation. The most recognised comprises first the pickup of the CNT–antigen conjugate by DCs, which transfer the antigen peptides to naive T cells for activation. To this work, a multitude of unique receptors exists on the surface of DCs, which naturally serve as recognition sites for activating specific immune cells. The movement of antigens to specific compartments for presentation in DCs is critical. In DCs, the lysosome-dependent pathway causes the antigen to break down into antigenic peptides (APs) inside lysosomes. APs are then loaded onto Class II major histocompatibility complex (MHC-II) molecules for presentation to CD4^+^ helper T cells. On the other hand, MHC-I molecules display cytosolic antigens to activate CD8^+^ T cells and trigger cytotoxic T lymphocyte (CTL) responses [232]. Among cytokines, TNF-α provides chemical signals to the cancer cells, causing inflammation and cell death [233]. Activated T cells and natural killer (NK) cells release IFN-γ, which activates macrophages and improves antigen presentation [234]. Other cytokines, such as IL-15 and IL-12, activate and stimulate proliferation and expansion of NK cells and other antitumour immune cells, including CD8^+^ T cells [235] (Figure 7).

The subsequent Table 11 collects studies where CNTs were utilised to synthesise CNT-based nanoplatforms for immune oncotherapy.

Immuno-based oncotherapy was experimented on by Xia et al. in vitro and in vivo, using MWCNT-based nano-delivery systems loaded with unmethylated CpG moieties, an oligodeoxynucleotide, and H3R6 polypeptide (MHR-CpG) for treating prostate cancer [241]. Authors observed enhanced biocompatibility, endosomal TLR9 targeting, and improved immunogenicity of CpG in both the humoral and the cellular immune pathways. An increase in the expression of CD4^+^ T-cells, CD8^+^ T-cells, TNF-, and IL-6 was detected. When tested in vivo in RM-1 prostate tumour-bearing mice, the nanocomposite was demonstrated to be capable of delivering the immune therapeutics to both the tumour site and to lymph nodes, thus inhibiting prostate cancer growth. Hassan et al. used antigen-bearing MWCNTs to deliver immunoadjuvants such as cytosine-phosphate-guanine oligodeoxynucleotide (CpG), anti-CD40 Ig (CD40), and ovalbumin (OVA) antigen to trigger an immune response against OVA-expressing cancer cells. When tested in vitro and in vivo, the MWCNT-based nanoplatform caused dramatically high OVA-specific T cell responses in vitro and in C57BL/6 mice. Co-loaded OVA antigen, CpG, and anti-CD40 Ig prevented the proliferation of OVA-expressing B16F10 melanoma cells in pseudo-metastatic subcutaneous or lung tumour models [159]. Furthermore, SWCNTs realised the efficient delivery of CpG in CX3CR1GFP mouse models, without toxicity to normal cells, and increased the production of proinflammatory cytokines by primary monocytes. Surprisingly, a single intracranial injection of low-dose CNT-CpG removed intracranial GL261 gliomas in half of the tumour-bearing animals via activation of NK and CD8^+^ cells and protected the surviving mice from the recurrence of intracranial cancer [242]. Oxidised MWCNTs (ox-MWCNTs) bearing COOH groups were prepared by Radzi et al. and tested for treating breast cancer in EMT6 model mice, associated with PTT. The combined therapy resulted in full cancer eradication and in a substantial increase in the mice’s median survival rate. Additionally, MWCNT-based nanocomposites increased the infiltration and maturation of DCs, CD8^+^, CD4^+^ T cells, macrophages, and NKs in tumours treated with ox-MWCNTs–hypothermia combination therapy [243].

Wilms’ tumour protein (WT1) is a protein upregulated in many human leukaemias and cancers. Villa et al. covalently attached WT1 ligands onto soluble SWCNT architectures, achieving SWCNT–peptide nanoplatforms, which were speedily absorbed by dendritic cells and macrophages in vitro. Immunisation of BALB/c mice with SWCNT–peptide nanocomposite and immunological adjuvants provoked specific IgG responses versus the peptide [244].

Fadel et al. utilised SWCNT bundles in the presentation of T-cell-activating antibodies to evoke immune responses in target tumours. SWCNT bundles delivered anti-CD3 T-cell-stimulating antibodies with high local concentrations, resulting in powerful activation of T cells, thus demonstrating that SWCNT bundles constitute a unique model for the effective activation of lymphocytes, with implications for fundamental science and clinical immunotherapy [245].

Functionalized bundled SWCNTs (*fb*-SWNTs) have been demonstrated to be efficient antigen-presenting substrates and were used by Fadel et al. to absorb T cell antigens and CD3 and CD28 costimulatory ligands. The achieved nanoplatforms were used to treat splenocytes obtained from the spleens of C57BL/6 mice. The adsorption of T-cell-stimulating antibodies improved both the kinetics and amount of T-cell activation, thus supporting the utilisation of chemically processed nanotube bundles in clinical applications requiring the presentation of artificial antigen [246].

Later, the same authors developed a simple yet robust technique of noncovalently attaching the T cell stimulus (MHC-I) to CNT substrates to avoid undesired denaturation effects. They used the achieved nanocomposite to treat OT1 mice, observing increased antigen-specific T cell responses, which were 3-fold higher than in the control [247].

Also, Burkert et al. developed AuNPs-bearing nitrogen-doped nitrogen nanotube cups (Au-NCNCs), which demonstrated the capability in entrapment and target delivery of paclitaxel in the tumour site. Au-NCNCs altered the TME, reduced tumour growth rate, and counteracted immunosuppressive macrophages [248].

Furthermore, it is dutiful to remember that the needle-like shape of CNTs, which allows them to penetrate cellular membranes, causing damage, can also trigger inflammatory responses that may lead to harm in both animals and humans. MWCNTs revealed high phagocytic activity towards undifferentiated HL60 cells and cytotoxic effects on differentiated HL60 cells [249].

Due to a lack of critical clinical evidence, the exact mechanisms by which CNTs can harm humans and animals remain unclear. On the other hand, many studies suggest that proper modifications can lower the hazardous effects of CNTs, making them eligible for applications in the biomedical field. Scientists in different fields concerning CNTs should conduct extensive research in collaboration to address concerns surrounding CNTs’ safety and to enhance their credibility. Looking ahead, researchers are expected to develop innovative synthetic methods or create novel composite materials to improve cancer treatment outcomes and enhance human health. 

## 7. Carbon Nanotubes Application in Cancer Diagnosis

**Figure 8:**
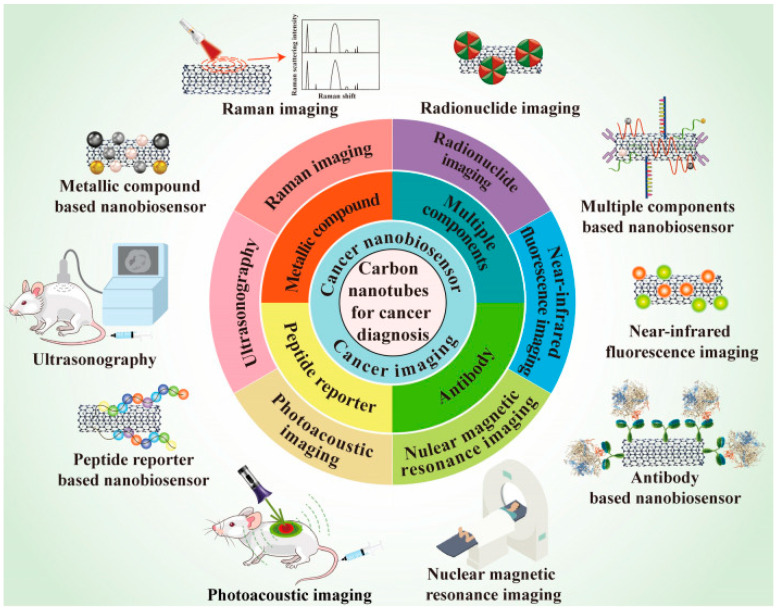
An overview of the contributions of CNTs to cancer diagnosis. The image has been reproduced by an open access article [250]. Reproduction is licensed under a Creative Commons Attribution 4.0 International License, which permits use, sharing, adaptation, distribution, and reproduction in any medium or format. To view a copy of this licence, visit http://creativecommons.org/licenses/by/4.0/ (accessed on 6 July 2025). The Creative Commons Public Domain Dedication waiver (http://creativecommons.org/publicdomain/zero/1.0/, accessed on 6 July 2025) applies to the data made available in this article, unless otherwise stated in a credit line to the data.

Cancer diagnosis aims at examining and detecting the aetiologies and related symptoms concerning various types of cancer, using modern technologies. Due to their nonpareil properties, CNTs are increasingly attracting interest also in this field. The application of CNTs in various cancer imaging, such as Raman imaging, nuclear magnetic resonance imaging (NMRI), ultrasonography (US), photoacoustic imaging (PAI), radionuclide imaging (RNI), near-infrared fluorescence imaging (NIR-FI), as well as their use to engineer cancer nano-biosensors, as schematized in Figure 8 and summarised in Table 12, are the topics of this section.

CNTs can provide multifunctional bio-probes with several unprecedented properties, including strong absorbance in NIR, good resonance Raman scattering, and high modifiability, thus representing excellent materials suitable for cancer imaging, with research and clinical prospective in cancer diagnosis.

### 7.1. Raman Imaging

The radial breathing model (RBM) and tangent G-module (TGM) are unique vibrational features of CNTs, which can be detected by a Raman microscope [267]. SWCNTs doped with oxygen, bearing epoxide groups, and modified with PEG (o-SWNTs@PEG) were engineered by Sekiyama et al. The achieved nanocomposites were created to serve as over-thousand- nanometre (OTN)-NIR fluorescent probes to investigate the time-dependent change in OTN-NIR fluorescence images of colon-26 cancer cells [251]. Upon administration of the probes to colon-26 cancer cells, their distribution in cells was studied using Raman microscopy, observing Raman signals on the fifth day from the first administration. Since noble metals on the surface of CNTs improve their Raman signals [268], Wang et al. enriched the surface of PEG@CNTs with gold or silver, thus improving the Raman scattering (SERS) effect of pristine CNTs. The use of noble metal-modified CNTs rather than that of non-modified PEG@CNTs allowed the acquisition of the Raman images under NIR radiation in remarkably minor time [252].

### 7.2. Nuclear Magnetic Resonance Imaging

Nuclear magnetic resonance imaging (NMRI) is a non-invasive imaging technique that does not use ionising radiation, thus not being risky for the human body. It provides the original 3D cross-section images of a tissue or organ and is of paramount help in medical imaging [269]. Since CNTs can be used as *T*_2_ spin dephasing contrast agents, they are utilized to enhance nuclear magnetic resonance imaging (NMRI) in clinics [270]. Yan et al., by the non-covalent association of the NGR (asparagine-glycine-arginine) peptide with DOX and CNTs bearing NMR contrast agent, namely Gd-DTPA, prepared a new nanocomposite theragnostic for both detecting the tumour by NMRI and anticancer therapy [253]. Zhang et al. synthesised a multimodal nanoplatform based on MWCNTs (FA@GdN@CQDs-MWNTs/DOX) using gadolinium NPs (GdN), magneto-fluorescent carbon quantum dots (CQDs), and folic acid (FA) [254]. In vitro targeting NMRI experiments revealed that FA-@dN@CQDs-MWNTs worked as excellent T1 contrasting agents, overstating the longitudinal proton relaxation process. As confirmation, in vivo assays showed that the NMR signal at the tumour site was positively enhanced after intravenous administration of FA@GdN@CQDs-MWNTs.

### 7.3. Ultrasonography

Ultrasonography is a low-cost and intrinsically safe diagnostic imaging technique [271]. CNTs are nanomaterials particularly eligible for ultrasonic imaging, since during ultrasonography, they are capable of producing high signals that can be detected by a contrast-enhanced ultrasound imager. Saghatchi et al. prepared multi-functionalized (*mf*) MWCNTs by their modification with both magnetic Fe_3_O_4_ and gold NPs (*mf*-MWCNT@AuNPs) for simultaneous cancer imaging and therapy [255]. This nanocomposite, when applied at various concentrations, exhibited a notably high contrast. Delogu et al. modified MWCNTs using azomethine ylides to improve their biocompatibility and applied the achieved nanocomposite (ox-MWNT-NH3^+^) in ultrasonography [256], observing a strong, long-lived, and high-quality ultrasound signal after sonication treatment.

### 7.4. Photoacoustic Imaging

In photoacoustic imaging (PAI), upon the irradiation of cells, tissues, and/or organs by a pulsed laser, the luminous energy is absorbed, converted into ultrasonic waves by the thermal expansion of tissues and organs, and detected by sensors [272]. Since CNTs possess strong NIR absorption and deep tissue penetration, they could serve as ideal contrast agents for PAI, whose signal can be recorded by photoacoustic microscopy [273]. Using PAI technology in cell imaging, Avti et al. detected, localised and quantified the content of CNTs in different tissue samples, thanks to signals that were clearly understandable and stable due to the potent NIR absorbance of CNTs [257]. Wang et al. engineered a multifunctional MWCNT-based probe encompassing the RGD peptide, a silica coating, and Au nanorods (RGD@sGNR@MWNTs) endowed with active targeting ability to be used for the in vivo PAI of gastric cancer [258]. After intravenous administration of the RGD@sGNR@MWNTs probe, the treated mice were analysed by an optoacoustic imaging system, evidencing that RGD@sGNR@MWNTs precisely targeted the tumour site and provided strong photoacoustic imaging effects.

### 7.5. Radionuclide Imaging

Radionuclide imaging (RNI) is an imaging technology that exploits radioactive isotopes such as ^111^In, ^131^I, ^64^Cu, and ^86^Y, which, upon injection into the body, are adsorbed by human tissues and organs, thus functioning as radiation sources in vivo emitting γ-rays during their decay process [274,275]. Such rays can be detected by nuclear detection devices, which provide the distribution density of the radioactive isotopes in vivo. RNI is endowed with deep tissue penetration, negligible limitation, and high sensitivity [276]. The organ biodistribution of various functionalized (*f*)-MWCNTs in vivo was studied by Wang et al., who used MWCNTs radio-labelled with ^111^In to empower easy in vivo single photon emission computed tomography/computed tomography (SPECT/CT) imaging [259]. Zhao et al. modified SWCNTs with polydopamine (PDA) and PEG, achieving a nanoplatform (SWNTs@PDA-PEG), which was further labelled with ^131^I for the subsequent RNI [260]. After in vivo administration, the tumour tissue distribution of ^131^I@SWNTs@PDA-PEG was detected by a gamma counter.

### 7.6. Near-Infrared Fluorescence Imaging

Near-infrared fluorescence (NIR-F) imaging, having an NIR biological wavelength window in the range of 780–1700 nm, is an appealing and fast-progressing imaging technique, extremely useful for cancer diagnosis. CNTs possess clear optical absorption and intrinsic fluorescence in the above-mentioned range, thus being promising materials for application in NIR-F imaging during cancer diagnosis, whose images are usually acquired using an in vivo imager [277,278]. Since SWCNTs possess higher absorbing properties, stronger optical absorption, better E11 optical transitions, and less photobleaching than MWCNTs, they are more appropriate for NIR imaging than the others [279]. Ghosh et al. developed an M13-stabilised SWCNTs probe, which exhibited in vivo precise targeting of tumour nodules expressing secreted protein, acidic and rich in cysteines (SPARCs) [261]. Second-window NIR light (NIR-II) was used in this study as the fluorescence source to avoid optical scattering and gain a deeper tissue penetration effect during NIR-F imaging. The diagnostic result achieved using this NIR2-emitting M13@SWCNTs probe evidenced outstanding signal-to-noise performance and high specificity towards in situ ovarian tumours and tumour nodules present on the surfaces of other peritoneal organs. Welsher et al. [262] combined SWCNTs with phospholipid-PEG (PL@PEG), achieving a biocompatible nanoplatform (SWCNTs@PL@PEG) with low toxicity levels and enhanced stability. Upon its administration in live mice and the application of an InGaAs camera, high-resolution intravital tumour vessel images were acquired.

### 7.7. CNTs in Nano Biosensors

Biosensors have been studied starting from the year 1962 [280], and recently, they are considered very attractive tools, mainly due to their easy application, fast response, and low cost [281]. Nano-biosensors (NBSs) are biosensors consisting of both biological recognition elements and nanomaterials, sized 1–100 nm [282,283]. The unparalleled electrical conductivity, excellent electrocatalytic properties, high stability, slow oxidation kinetics, and good modifiability of CNTs render them a class of nanomaterials with great potential for applications as NBs in cancer diagnosis [284].

### 7.8. CNTs Combination with Metallic Nanoparticles

Gold and silver nanoparticles (NPs) (AuNPs and AgNPs) are used to modify CNTs to allow the construction of NBs. Rawashdeh et al. reported on AuNPs@MWCNTs, which worked well as NBs, demonstrating easy and improved detection of micro ribonucleic acid named miR-21, which is of paramount importance in the early diagnosis of pancreatic cancer [263]. AuNPs@MWCNTs had a limit of detection (LOD) as low as 3.68 femtomolar (fM) using the source measure unit (SMU).

### 7.9. CNTs Combination with Antibody

Nano-immunosensors (NISs) based on antibodies are a particular type of NBSs used in cancer diagnosis. Osteopontin (OPN) is a biomarker that is used to detect prostate cancer cells, and its levels are important to predict the survival time of patients bearing prostate cancer [285,286]. To overcome the issues that affect the traditional method used for the measurement of OPN (ELISA assay), Sharma et al. covalently combined OPN monoclonal antibodies onto the SWCNTs@COOH surface, achieving an NIS to determine OPN for prostate cancer diagnosis [264]. AntiOPN@SWCNTs@COOH exhibited high specificity to only OPN, a broad detection range, and a low LOD.

### 7.10. CNTs Combination with Peptide Reporter

Detecting CDK1 peptide in tissues and organs could be of great help in the early diagnosis of tumours. Furthermore, diagnostic methods using traditional BSs for the detection and quantification of activities of CDK1 pose great difficulties due to the lack of sensitivity and specificity [287]. Aiming at overcoming these issues, functionalized MWCNTs further modified with a fluorescent peptide reporter specific to CDK1 were developed for the sensitive and fluorescence-based quantification of CDK1 activity via fluorescence imaging [265]. The as-prepared NBS provided both easy detection of CDK1, reported the enzymatic activity of CDK1, and detected the CDK/cyclin activity in numerous cancer types, usually associated with poor prognosis.

### 7.11. CNTs Combination with Multiple Modifications

Multiple modifications on CNTs can further improve the diagnostic effects obtained using the NBSs engineered via a single modification. Mahmoodi et al. developed an rGO@MWCNTs/_L_@Cys@AuNPs multifunctional nanocomposite and enriched its surface with a single-strand DNA (ssDNA) probe as an electrochemical DNA biosensor to diagnose HPV-18 [266]. rGO@MWCNTs/_L_@Cys@AuNPs@ssDNA demonstrated high selectivity and sensitivity to HPV-18 towards both the extracted DNA from HPV-18 patients and the synthetic one. Overall, this constructed rGO@MWCNTs/_L_@Cys@AuNPs@ssDNA NBS represents an essential tool for the early, easy, rapid, and accurate diagnosis of HPV-18. Table 13 summarises other relevant case studies on the application of CNTs as sensors for early diagnosis of cancer.

## 8. Clinical Translatability and Challenges Concerning CNTs

CNMs are unprecedentedly effective tools with nonpareil properties in cancer theragnostic. Furthermore, the gap between laboratory research and clinical transformation still needs a long time and more in-depth research. The clinical translatability (CT) of carbon nanomaterials (CNMs), including CNTs, is a relevant issue that must be considered to reach the goal of ameliorating conditions and life expectancy of tumour-bearing patients. Furthermore, many problems, such as their toxicity, low biodegradability, unclear metabolism, scalability of production, and regulatory hurdles, remain unsolved and should be addressed before their successful translation into clinical practice. Concerning CNTs’ toxicity, in terms of cytotoxicity, genotoxicity, reprotoxicity, and toxicity to specific organs, an extensive discussion is available in our recent works [55,56,76]. Briefly, CNMs’ toxicity, mainly dependent on their sizes, structure, shape, surface chemistry, and on the type of cells targeted [305], should be taken into great consideration when they have been developed for clinical biomedical applications. Research should be focused on finding methods to prepare ideal CNM structures without defects, impurities, and low cytotoxicity, mainly by tuning size and designing suitable decorations. To quicken their translation in clinical practice, their stability in physiological conditions, cellular uptake, biodistribution, and accumulation in different tissues and organs should be ascertained in advance. Their transformation and metabolic pathway in vivo, as well as their acute and latent toxicity, should be clarified [306]. To obtain the desired theragnostic goals, the delivery efficiency of CNMs of therapeutic agents specifically at the tumour site is of paramount importance. A suitable CNM size is pivotal for realising enhanced permeability and retention (EPR)-mediated passive tumour targeting [307]. Conversely, to achieve active targeting, a deep study of the receptors overexpressed specifically on the tumour cell surface is mandatory. Once CNM-based nanoplatforms capable of efficient therapeutics delivery are realised, the endogenous or exogenous stimuli should be fully exploited to accomplish controlled drug release. Unfortunately, many obstacles still exist, hampering the achievement of these goals, such as complicated design, boring synthesis, scarce synergistic functions, poor integrated efficiency, and ambiguous biological response. Also, while the antitumour properties of CNMs have been extensively demonstrated [308], their antineoplastic mechanism and their tumour proliferation and metastasis regulation machinery have not been systematically clarified. In this regard, before the direct clinical application of CNMs as anticancer drugs, their metabolic pathway, biological safety, and how they can be used as anticancer agents for cancer treatment to overcome the limitations of conventional chemotherapeutics should be studied and clarified. Moreover, because of hydrophobicity and structural stability, CNMs suffer from low solubility and dispersibility in aqueous media, poor miscibility, and a tendency to self-aggregate, which mostly limit their biological applications [309]. When CNTs are applied as drug delivery systems (DDSs), due to their stability, free CNTs survive in the body for a long time after drug dissociation, thus triggering secondary damage [310]. Problems that impede CNTs’ clinical application can be summarised as follows.

1. The mechanisms of action of CNTs on normal cells and tissues are not fully unveiled.
2. Potential toxic effects can occur due to their special structure.
3. The mechanisms of CNT-induced toxicity are not fully clarified.
4. The biodegradability of raw CNTs is too low because their hydrophobic properties prevent enzymes from approaching them, thus impeding enzymatic degradation.

To promote CNTs translatability in clinical practice, more labour is necessary to explore the best functional molecules for the CNTs modification to improve their biocompatibility, to avoid the toxic and risk factors, to enhance their solubility, and to create defect sites, which offer desired binding sites for enzymes and promote enzymatic degradation [311]. Collectively, although there is no clinical practice that has ever reported the application of CNMs as DDSs in cancer theragnostic, there are some clinical trial cases that assessed the employment of carbon nanoparticles (CNPs) in lymphatic monitoring during colorectal cancer surgeries, lymph node collection in advanced gastric cancer, and lymph node biopsy of papillary thyroid carcinoma, confirming the unprecedented potential and promising future of CNMs and CNTs in cancer theragnostic [312,313,314]. The following Table 14 and Table 15 summarise two others clinical trials carried out to evaluate the possible applications of CNT-based nanocomposites in anticancer treatments and cancer diagnosis.

Other clinical trials regarded the application of CNTs to treat dental caries, Parkinson’s (available online at https://clinicaltrials.gov/study/NCT02873585, https://clinicaltrials.gov/study/NCT01246336, accessed on 26 May 2025), and cervical pain [318]. The assessment of the clinical observational studies reported in Table 14 focused on the potential for misleading selection of participants, measurement of exposures and outcomes, handling of missing data, and selective reporting of results. This comprehensive approach ensured a thorough examination of factors that could influence the results of the study, from the allocation of participants to the reporting of the outcomes. Each domain was rated as “low risk 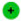
”, “high risk 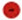
”, or “some concerns 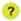
”, based on specific criteria [319,320]. This assessment process emphasised the importance of transparency and rigour. Using the Cochrane Risk of Bias, two reviewers independently assessed the risk of bias (Table 16).

A wide dissertation on the regulatory hurdles concerning the large-scale production and application of CNTs in several sectors is already available in previous papers [55,56]. Production and application of CNT-based nanocomposites on a large scale are mainly hampered by the prohibitive costs still required for their production and purification [55]. Years of research in industry and in academic laboratories concerning the development of automated systems for producing CNTs, possessing uniform and predictable properties, as well as reduced toxicity, have recently led to the development of a carbon copilot (CARCO), which is an artificial intelligence (AI)-driven platform integrating transformer-based language models, robotic chemical vapour deposition (CVD), and data-driven machine learning models [321]. Employing CARCO, Li et al. found a new titanium-platinum bimetallic catalyst for high-density horizontally aligned carbon nanotube (HACNT) array synthesis. This catalyst outperformed traditional ones, and through millions of virtual experiments, an unprecedented 56% precision in synthesising predetermined densities of HACNT arrays was achieved [321].

## 9. Interaction of CNTs with Biomolecules and Tactics to Reduce Their Toxicity

### 9.1. Interactions of CNTs with Biomolecules

It has been reported that unmodified CNTs can interact with biomolecules mainly via van der Waals forces and p-stacking of sp^2^ bonds, while modified CNTs can also exploit functional groups appositely introduced by post-synthesis reactions, including carboxylate or amino group moieties. In this case, CNTs can interact with biomolecules via hydrogen bonding, hydrophobic effects, covalent, electrostatic, and p-p stacking relations [322,323].

The interactions between CNTs and biomolecules have been studied using several analytical techniques. In particular, the bio-layer interferometry (BLI)-based biomolecular interaction assay was adopted to have notable information on kinetic binding (k_on_, k_off_, K_D_) between CNTs and biomolecules [324]. The binding of biomolecules to CNTs was investigated mainly using spectroscopy based on a subtraction method [325]. Furthermore, in the case of proteins at high concentration, such a method might have huge experimental errors and result in false positives.

CNTs have been shown to be able to interact well with proteins, peptides, nucleic acids, etc. Using ForteBio and Plexera methods, the authors demonstrated that the binding affinities of biomolecules to CNTs are dependent on their surface modifications [324]. In the Plexera assay, biomolecule binding was detected by surface plasmon resonance (SPR) technology, while in the Forte Bioassay, it was detected by Bio-Layer Interferometry (BLI)-based technology. Both assays showed that nonfunctional CNT (p-MWCNTs) could not bind to proteins effectively, whereas the carboxylate functional CNT (f-MWCNTs) bound to proteins extremely tightly with a very small off-rate, with its binding K_D_ to WGA at 4.6 × 10^−11^ M and FKBP12 at 3.2 × 10^−9^ M [324].

Since kinetic data of CNTs binding to biomolecules (proteins, peptides, nucleic acids, and small molecular drugs) were rare, Zhang et al. extended early kinetic studies [324], thus measuring and reporting additional k_on_, k_off_, and K_D_ values of f-MWCNT or p-MWCNT binding to various biomolecules.

According to results, f-MWCNT showed strong binding to random proteins such as KGA and FKBP52 with K_D_ of 3.2 × 10^−6^ M and 1.7 × 10^−5^ M, respectively, while p-MWCNT showed no binding to any of the same proteins.

Authors observed that the binding modes of f-MWCNT to various biomolecules are different. f-MWCNT interacted with proteins and DNA strongly and non-selectively. They were demonstrated to interact indifferently with any proteins, DNA, and lipids, thus being able to penetrate cell membranes directly [322] and to inhibit calcium channels [326]. Strong dose-dependent binding to random proteins such as BSA, WGA, and FKBP12 was observed [324]. The single-strand (ss)DNA with 20 base pairs also bonded very strongly to f-MWCNT with a very small off-rate. Notably, the random and tight binding of f-MWCNTs to DNA and proteins can inhibit DNA replication, transcription, translation, and cellular signalling, which can translate into in vivo cytotoxicity.

### 9.2. Tactics to Reduce CNT Toxicity

It has been established that CNTs can accumulate in the environment and human bodies, thus having serious noxious outcomes, which can also derive from the CNTs-evoked activation of toxic systems in the tissues of organisms that come into contact with them [76,327,328,329,330,331,332]. In this regard, several scientists have studied strategies to minimise such activations, mainly based on CNT surface modifications and functionalization [76,327,328,329,330,331,332]. Polyethylene glycol (PEG), C1q recombinant globular proteins, and biocompatible ingredients or molecules, which can improve CNTs’ solubility and dispersity in biological fluids, have been exploited to modify CNTs’ surfaces. Moreover, to reduce MWCNTs’ capacity to induce oxidative stress (OS), oxidative damage, inflammation, and immune-toxic effects, antioxidant natural molecules, such as curcumin or quercetin, have been experimented [333,334,335,336]. Also, to affect cell uptake, the CNT surface was functionalized with -COOH and -OH groups [337,338]. An effective purification of CNTs is essential to reduce their toxicity, often due to residual metals or catalysts, which are noxious to living organisms. In this regard, several advanced purifications workups have been studied to obtain highly pure, non-defective, and less toxic CNTs [339]. Furthermore, more biodegradable CNTs over time have been developed to reduce their persistence in the blood and reduce tissue toxicity [340]. Table 17 collects some important tactics suggested so far to lessen the possible dangerous outcomes that could come from extensive exposure to CNTs.

## Figures and Tables

**Table 1:** Examples of in vitro and in vivo applications of CNT-based nanocarriers associated with different types of anticancer therapies.

CNTs	Measures	Cell Line/Animal Models	Drug(s)	Concentration/Dose	Results	Refs
MWCNTs	L = 330 nm Ø = 30 nm	BM-MSCs, MDA-MB-231 CD44high, CD24low CSCs	Pt-NPs, PBI	100 µM	Cause cell cycle arrest, diminish drug resistance Impede DNA repair in breast CSCs.	[79]
CNTs	N.R.	Kunming mice	Span, PEG, FA Paclitaxel	350 mg/kg	Penetrate breast tumours Inhibited development, induced tumour cell death	[80]
SWCNTs	N.R.	HT-29	Porphyrin, PEG	25 mg/mL	No antitumour activity	[84]
MWCNTs	Ø = 17.1 ± 3.0 nm	C540 Male Balb/c mice	Poly pyrrole	250 mg/mL, 10 mg/kg	Concentration-dependent cytotoxicity under multi-step ultrasonic irradiation (8.9% cell viability for 250 mg/mL) 75% Necrosis and 50% TVR after 10 days of SDT	[81]
MWCNTs	Ø = 10 nm L = 5–15 µm	Male Balb/c mice	CREKA	MWNTs-PEG 2 mg/kg CMWNTs-PEG 4 mg/kg	6.4 Times ⬆ accumulation in tumour tissue Xenograft eradicated after 4 cycles of illumination	[82]
MWCNTs	Ø = 5–20 nm L = 1–10 µm	HepG2, HeLa cells C57BL/6J female mice	PEG-O-CNTs	0–1000 mL 1 mg/mL injected in tumour at 200 µL/cm^3^	⬆ Cell toxicity of PEG-O-CNTs, O-CNTs, pure CNTs Continuous-wave NIR laser diode (808 nm) for 10 min ⬆ TVR in animal group (PEG-O-CNTs)	[83]

L = length; Ø = diameter; PEG-O-CNTs = oxidised CNTs conjugated to polyethylene glycol (PEG); FA = folic acid; Pt-NPs; PBI = polybenzimidazole; MTX = methotrexate; SDT = sonodynamic therapy; TVR = tumour volume reduction; PTT = photothermal treatment; ⬆ = higher; CSCs = cancer stem cells; BM-MSCs = bone marrow mesenchymal stem cells (BM-MSCs); Pt-NPs = platinum nanoparticles; CREKA peptide = linear polypeptide composed of 5 amino acids arranged by Cys-Arg-Glu-Lys-Ala.

**Table 2:** Functionalization of CNTs through various molecules and their applications as anticancer agents.

CNTs	Functionalizing Molecules	Effectiveness	Tumour Model	Biocompatibility Test	Refs.
SWNTs	PEG	⬆ Solubility, prevent particle aggregation, ⬇ side effects	Gastric cancer	⬇ Toxicity towards normal tissue	[91]
SWNTs/MWNTs	PEI	⬆ Solubility, homogeneity, dispersity, ⬇ particle size	Cervical cancer	N/A	[92]
		⬆ Positive charge to interact with siRNA			
SWNTs	HA	⬆ Stability in serum, overcome MDR	Ovarian cancer	No cytotoxicity to normal tissue	[93]
		Target CD44-overexpressing cancer cells		No drop in mice weight	
MWNTs	Chitosan	⬆ Solubility, ⬆ cell-penetrating ability, ⬇ toxicity	Breast cancer	⬇ Cytotoxicity	[94]
MWNTs	PLGA	⬆ Dispersity, ⬇toxicity, provide, tune the temporal release	Osteosarcoma	⬇ Cytotoxicity in normal cells	[95]
		Provide attachment sites for drugs			
SWNTs	CD33 mAB	Recognize and specifically target the GNM-CD33^+^ cells	Glioblastoma	N/A	[96]
SWNTs	IGF1R mAB/HER2 mAB	Target IGF1R and HER2 surface receptors	Breast cancer	⬇⬇⬇ In vitro toxicity in normal cells	[97]
SWNTs	RGD peptide	Target α_v_β_3_-expressing cancer cells	MM	⬇ Toxicity in vitro	[98]
SWNTs	EGF receptor	Active targeting ability, ⬆ uptake of drugs	HSC, NSC	⬇⬇⬇ Toxicity in vitro and in vivo	[99]

MDR = multidrug resistance; MM= malignant melanoma; HSC = head squamous carcinoma; NSC = neck squamous carcinoma; enhanced, improved, high, higher; low, lower, decreased, reduced; PEG = polyethylene glycol; PEI =polyethylene amine; IGF-1R = insulin-like growth factor receptor 1R; WS = water solubility; HER2 = human endothelial receptor 2; RGD = arginyl-glycyl-aspartic acid; EGF = epidermal grow factor; ⬆ = high, higher, improved, enhanced, increased; ⬇ = low, lower, decreased, reduced; ⬇⬇⬇ = significantly reduced, absent, eliminated.

**Table 3:** In vitro and in vivo experiments that used modified CNTs as drug and gene delivery systems for various chemotherapeutics and nucleic acids.

Drug/Gene	Modified	Cancer Type	In Vitro	In Vivo	CNTs	Therapeutic Outcome	Refs
Pt (IV) drug, PD	PL-PEG F	Testicular cancer	Ntera-2	N.R.	SWCNT	25 Folds ⬆ cytotoxicity	[110]
CP	Covalent amide linkage	Head, neck cancer	HNCC	N.R.	SWCNT	⬆ Effective than free CP and siRNA conjugate	[99]
CA	Interior filling	Bladder cancer	EJ28	N.R.	SWCNT MWCNT	Controlled release properties	[111]
OP	PEG600 F	Colorectal cancer	HT29	N.R.	MWCNT	Delayed cytotoxic activity	[112]
DOX	PEGylation F	N.R.	Ascites Sarcoma 180	N.R.	SWCNT	⬆ Retention of drug in situ	[113]
						⬇ Effect on other tissues	
	Not-F	Breast cancer	MCF-7	N.R.	MWCNT	⬆ Cytotoxicity	[114]
	P-gp F	Leukaemia	K562	N.R.	SWCNT	⬆ Cellular uptake	[115]
						23 folds ⬆ cytotoxicity	
PTX	PEGylated F	Lung cancers	A549, NCI–H460	N.R.	SWCNT	⬆ PTX activity	[116]
		Ovarian cancer	OVCAR3	N.R.	SWCNT	Chemosensitizer, ⬆ cell death	[117]
	Poly citric acid F	Lung, ovary cancer	A549, SKOV3	N.R.	MWCNT	⬆ Potency	[118]
	PEGylated F	N.R.	N.R.	Murine 4T1	SWCNT	⬇⬇⬇ Tumour growth	[119]
						10 folds ⬆ uptake	
SB-T-1214, TAXO	Biotin F	Lung cancer, leukaemia	L1210FR, L1210 W138	N.R.	SWCNT	⬆ Target due to biotin	[120]
GEM	Folic acid F	Breast cancer	MCF-7 cells	N.R.	MWCNT	⬆ Cytotoxic than free GEM	[121]
	PEGylated F	Pancreatic cancer	N.R.	BxPC-3–B/c		⬇ Metastatic lymph nodes	
HIF-1α siRNA	Cationic F	N.R.	N.R.	MiaPaCa	SWCNT	⬇⬇⬇ Tumour growth	[122]
				2/HRE MM			
Anti-EGFP siRNA	PEI and Pyridinum F	Lung cancer	H1299	N.R.	MWCNT	⬆ Cytotoxicity	[123]
Cyclin A_2_ siRNA	Amine (NH_3_^+^) F	Leukaemia	K562	N.R.	SWCNT	⬇ Cell proliferation and	[124]
						apoptosis	
PLK 1 siRNA	NH_3_^+^ F	Lung cancer	Calu6	LCXGM	MWCNT	⬇⬇⬇ Tumour	[125]
siNEG siRNA	Amidation with PUT SPERD, SPER	Lung cancer	A549	N.R.	SWCNT MWCNT	⬇ Tumour proliferation	[126]
Bcl9l siRNA	Aptamer and Pyr-PEI F	Breast cancer	MDA-MB-231	N.R.	SWCNT	Targeted silencing activity	[127]
GAPDH siRNA	PEI F	Cervical cancer	HeLa	N.R.	SWCNT	Induced cell death	[92]
siTOX, siNEG	Cationic DD F	Cervical, lung cancer	HeLa, A549	N.R.	MWCNT	⬇ Toxicity, effective delivery Gene silencing capabilities	[128]
siRNAs							
PLK-1 & siTOX siRNAs	NH_2_-F or cationic liposomes	Breast, cervical, lung, melanoma, prostate, renal, kidney cancers	Calu6, A549, DU145	LCXGM	MWCNT	Apoptosis, ⬇⬇⬇ tumour	[129]
			C-33A, MCF-7, HEK293 HeLa, NIH 3T3 B16F10				
PLK1 siRNA	NH^4+^ and Guanidium DD	Cervical cancer	HeLa	N.R.	MWCNT	Significant silencing of PLK-1	[130]
RNAi	PEHI-pHSP-shT,	Breast cancer	MCF-7	Nude mice	SWCNT	Gene knockdown, ⬆AA	[131]
pDNA	Cationic glyP F	Cervical cancer	HeLa	N.R.	SWCNT	Biocompatible, ⬆ transfection *	[132]
p53 plasmids	Ethylenediamine F	Breast cancer	MCF-7	N.R.	SWCNT	Induced apoptosis	[133]
pDNA	Ammonium F	Cervical cancer	HeLa, CHO	N.R.	SWCNT & MWCNT	First reported gene carrier	[134]
GFP gene	PAMAM	Cervical cancer	HeLa	N.R.	MWCNT	⬆ GFP gene transfection	[135]
dsDNA	Multi-F	Epithelial cancer	Mouse ovarian epithelial	N.R.	SWCNT	Targeted drug delivery	[136]
miRNA	PEI-g-GNR	Cervical cancer	HeLa	N.R.	MWCNT	New nonviral vector for in situ detection	[137]
dsODN-NF-kB	Carbodiimide F	Cervical cancer	HeLa	N.R.	SWCNT	Targeted successful deliver	[138]
ANT *c-myc* ODNs	PAMAM	Breast, liver cancer	MCF-7, MDA-MB-435, HepG2	N.R.	MWCNT	⬆⬆⬆ Gene efficiency	[139]
MUC-1 Apt	Carboxylate F	Breast, lung cancer	MCF-7, Calu-6	N.R.	MWCNT	Aptamer successful integration	[140]

⬆ = high, higher, improved, enhanced; ⬇ = low, lower, decreased; reduced; ⬆⬆⬆ = strongly enhanced; ⬇⬇⬇ = strongly reduced; suppressed; N.R. = not reported; CP = Cisplatin; CA = carboplatin; OP = oxaliplatin; DOX = doxorubicin; PTX = paclitaxel; TAXO = taxoid; GEM = gemcitabine (GEM); EGPF = enhanced green fluorescent protein; CyA2 = cyclin A2; PLK 1 = polo-like kinase 1; GAPDH = glycerinaldehyd-3-phosphat-dehydrogenase; hTERT = human telomerase reverse transcriptase; *p*DNA = plasmid DNA; GFP = green fluorescent protein; dsDNA = double-stranded; dsODN = double-stranded oligodeoxynucleotides; NF-kB = nuclear factor ‘kappa-light-chain-enhancer’ of activated B-cells; MUC-1 = mucin-1 protein; ANT = antisense; F = functionalization; * = lipofectamine 2000; PAMAM = poly-amidoamine; glyP = glycol-polymer; PEI = poly-ethylenimine; PEHI = polyetherimide; pHSP-shT = Hsp70B′-promoter-driven RNAi (RNA interference) vector. DD = dendron; Pyr = pyrazine; SPER = spermine; SPERD = spermidine; PUT = putrescine; PD = prodrug; LCXGM = xenografted mice; B/c = BALB/c mouse; TBM = tumour-bearing mice; MM = mouse model; AA = antitumor activity.

**Table 4:** Improved biocompatibility and anticancer efficiency of several modified CNT-based carriers used to deliver in vitro and in vivo anticancer drugs and nucleic acids to treat various tumours.

CNTs	Functionalised Molecules	Effectiveness	Tumour Model	Biocompatibility Test	Refs.
MWCNTs	FA	Targeted and pH-sensible release of DOX	N.R.	N.R.	[141]
SWCNTs	PEG-10–10%PEI/pDNA	Allowed dosage 58-fold ⬇ conventional IC_50_	AGS and L929 *	⬇ Toxicity	[91]
		⬆ WS, ⬆ biological fluids dispersity			
SWCNTs	PEG-PCL and PEG-PCL-PEI	Faster acidic DOX release; ⬆ anticancer efficacy, ⬆ WS	MCF-7 *	⬆ Biocompatibility	[142]
SWCNTs	ALG, CHI, FA, DOX	Precise magnetic field-dependent CNT vibration causing cancer cell membrane destruction and DOX release	Lung cancer *	⬆ Biocompatibility	[143]
SWCNTs	COOH-, PEG- PEI- DOX	Faster acidic DOX release, ⬆ Antitumour activity	MCF-7 *	⬇ Toxicity to normal cells	[100]
		⬆ Dispersibility, ⬆ Affinity for tumour cells.			
SWCNTs	PEI-BET-DOX and SUR siRNA	⬆⬆⬆ pH-responsive lysosomal escape of siRNA	HeLa, A549 *^,^**	⬇ Toxicity to normal cells	[144]
		⬆ Antitumour properties			
SWCNTs	Chim/PEI/5-FU/CNT	⬆⬆⬆ Tissue penetrability, ⬆⬆⬆ DL, CT allowed	Gastric cancer *	⬆ Biocompatibility	[145]
		⬇ Invasion and proliferation, apoptosis in 5-FU-RCCs			
SWCNTs	II-NCC	⬆⬆⬆ SWCNTs water dispersion; ⬆ Anticancer effects of CAP	Caco-2 *	⬆ Biocompatibility	[146]
MWNTs	iRGD-PEI-MWNT-SS-CD/pAT_2_	⬆ WS and complexation capacity by PEI, ⬇⬇⬇ Tumour growth	A549 *^,^**	⬆ Biocompatibility	[147]
		Target smart release in TRE by SS			
SWCNTs	PEG and PEI	⬆ WS, ⬆ Cytotoxicity	AMJ13 *	⬇ Cytotoxicity to normal cells	[148]

* In vitro experiments; ** in vivo experiments; DL = drug loading; SS = cystamine; FA = folic acid; PEG = polyethylene glycol; PEI = polyethylene imine; pDNA = plasmid DNA; PCL = polycaprolactone; ALG = sodium alginate; CHI = chitosan; DOX = doxorubicin; BET = betaine; SUR = surviving; siRNA = silencing RNA; Chim = chimera; 5-FU = 5-fluorouracil; II-NCC = N, carboxy cellulose II; iRGD = tumour-homing peptide; CD = candesartan; pAT_2_ = plasmid AT_2_; CAP = capecitabine; ⬆ = improving, improved, augmented, high, higher; ⬇ = low, lower, decreased; reduced; ⬆⬆⬆ strongly enhanced, increased; suppression; WS = water solubility; TRE = tumour-reducing environment; CT = combination therapy; RCCs = resistant cancer cells; ⬇⬇⬇ = suppressed.

**Table 5:** Some examples of application of CNT-based DDSs to deliver the transported material to specific intracellular sites and TME constituents.

TT/Modulation	DDS	TM	Model	Effectiveness	Refs.
Cytoplasm	SWNT-PS/siRNA	PTT, GT	Hela	⬆ Gene transport capacity, ⬆ antitumour activity Controlled gene release	[156]
Cytoplasm	SWNT-CY7-IGF-1Ra	PTT, IT	ASPC-1, BXPC-3 PANC-1 SW1990 *	Precise tumour-targeting therapy, ⬆ body weight ⬆ Survival rate of tumour-bearing mice	[157]
MITO	SWNTs-PL-PEG-NH_2_	TAT	H22 liver cancer	Selective ⬇⬇⬇ of tumour mitochondria Tumour cell apoptosis	[158]
CSCs	N.R.	IT	MDA-MB-231 breast cancer	Realise active targetability towards breast CSCs	[154]
Antigen-presenting cells	MWNTs-CpG-αCD40-OVA	IT	B16F10 melanoma	⬆ Co-loading ability of OVA, CpG, and anti-CD40 Ig ⬇ TG and metastasis	[159]
TV	iRGD-PEI-MWNT-SS-CD/pAT_2_	CT	A549 lung cancer	⬇⬇⬇ TG and neovascularization	[147]
Cytoplasm	SWCNT-FA/DOX	CT, NIR-I	Lung cancer A549	External-stimuli-dependent DOX release, ⬆ anticancer effects	[160]
Cytoplasm	SWNT-HIF-1α/siRNA	GT	Pancreas	Transfected tumour, activated RNAi response, ⬇⬇⬇ TG	[122]
Cytoplasm	Ox-DWNT/siRNA	GT	Prostate	Prevent surviving creation and induce apoptosis by the release of siRNA into cytoplasm	[161]
Nucleus	PEG/SWNTs/DOX	PTT + CT	Breast	⬆ Delivery effectiveness, ⬆ DOX localisation/accumulation inside nucleus, ⬆⬆ killing of cancer cells	[162]
Nucleus	SWNTs-carrier	CT	Colorectal	Targeted therapy, regulated medication release	[163]
Nucleus	*f*-SWNTs/p53	GT	Breast	⬆ Transport and targeting gene into the nucleus, apoptosis	[133]
MITO	MWNT-Rho (PtBzt + BP)	CT	Ovarian	Still ⬇ selective	[164]
MITO	PEG/CNTs/ABT737	CT	Lung	⬆ Cell targeting to mitochondria, apoptosis	[165]
MITO	P-D-CS-CNTs	PTT	Bladder	⬆ Mito targeting with damage, ROS burst, cancer cells death	[166]
MITO	PL-PEG-SWNT	PAT	Breast	MITO malfunction, MITO outer membrane permeabilization	[167]
ECM	MWNTs	PTT	Epidermoid	⬇⬇⬇ Tumours, ⬇ volume, collagen destruction, cell damage	[168]
CCSs	SWNT-Raw, SWNT-COOH	CT	Osteosarcoma	Activated TGFβR1, ⬇⬇⬇ its signalling, ⬇ OSCs population	[169]
TV	DOX/CD-CNT, CUR/CD-CNT	PTT + CT	Hepatocellular	⬆ DEE and achieved sustained release of both drugs	[170]
TV	iRGD-PEI-MWNT-SS-CD/pAT2	CT	Lung	⬆ CU, TE, ⬇⬇⬇ angiogenesis, ⬇⬇⬇ TG	[147]
PD-1/PD-L1	Rg3-CNT	IT	Breast	Inhibit PD-1/PD-L1 axis and the TNBC cell growth	[171]
IMs	MWNTs-DOX and MWNTs-CpG	IT + CT + PTT	Melanoma	⬇⬇⬇ TG, ⬆ CD4^+^, CD8^+^ T cells, ⬆ TAM shifting ⬇ Tregs in TME	[172]

TT = therapeutic target; DDS = drug delivery system; GT = gene therapy; IT = immunotherapy; CT = chemotherapy; * pancreatic cancer cells; CD40 = clusters of differentiation 40; CpG = cytidine-phosphate-guanosine; CSCs = cancer stem cells; IGF-1Ra = insulin-like growth factor-1Ra; MWNTs = multi-wall carbon nanotubes; OVA = ovalbumin; pAT_2_ = plasmid angiotensin II type 2 receptor; PEI = polyetherimide; PEG = polyethylene glycol; PL = peptide lipid; PTT = photothermal therapy; SWNTs = single-wall carbon nanotubes; SS = disulphide bond; ⬆ = high, higher, improved, enhanced; ⬇ = low, lower reduced; ⬆⬆ = very high, very enhanced; ⬇⬇⬇ = suppression, inhibition; MITO = mitochondria; Rg3 = ginsenoside component of puffed ginseng with anti-cancer activities; RGD = arginyl-glycyl-aspartic acid; ECM = extracellular matrix; CCSs = cancer stem cells; IMs = immune cells; TV = tumour vasculature; TME = tumour microenvironment; DDS = drug delivery system; SOR = sorafenib; HIF-1α = hypoxia-inducible factor-1 alpha; TM = therapeutic modality; PTT = photothermal therapy; PAT = photoacoustic therapy; Ox-DWNT = oxidized double-walled carbon nanotubes; f-SWNTs-p53 = functionalized SWCNTs/plasmid 53 complexes; Rho/(PtBzt + BP) = Rhodamine-110/ Platinum (IV) prodrug+3-bromopyruvate; ABT737 = selective small molecule B-cell lymphoma 2 (Bcl-2) Homology 3 (BH3) mimetic; CS = chitosan; PL = phospholipid; NCs = normal cells; CUR = curcumin; DOX = doxorubicin; CD = candesartan; SS = cystamine; EI = epithelial injury; TG = tumour growth; DEE = drug entrapment efficiency; CU = cellular uptake; TE = transfection efficiency; TGFβR1 = TGFβ type I receptor; TAM = tumour-associated macrophage; TV = tumour vasculature; NIR-I = near infrared irradiation.

**Table 6:** Application of modified CNTs as nanocarrier for the tumour microenvironment (TME)-responsive release of anticancer drugs.

CNTs	Functionalizing Molecules	Effects	TM	Biocompatibility	Refs.
MWNT	DOX, Cis, PEG, FA	Stopped Cisplatin release, pH-sensitive release, synergic antitumour impact	L929, MCF-7	⬆ Biocompatibility	[178]
SWCNTs	DOX-CS/PNIPAAm	⬆ Release at 40 °C than 25 °C, at pH 5.0 than pH 7.4	HeLa	⬆ Biocompatibility	[180]
		⬆ dual stimuli DOX release (NIR + AE of TME)		⬆ Biocompatibility	
SWNTs	PEG	⬆ WS, prevent PA	Gastric cancer	⬇ Toxicity to NTs	[91]
SWNTs/MWNTs	PEI	⬆ WS, homogeneity and dispersity, ⬇ particle size, ⬆ PC, ⬆ interaction with siRNA	Cervical cancer	N/A	[92]
SWNTs	HA	⬆ SS, ⬆ target CD44-overexpressing cancer cells, overcome MDR	Ovarian cancer	No cytotoxicity to NTs	[93]
MWNTs	CS	⬆ Water solubility and cell-penetrating ability	Breast cancer	⬇ Cytotoxicity	[94]
MWNTs	PLGA	⬆ Dispersity, ⬆ attachment sites, tuned temporal release	Osteosarcoma	⬇ Cytotoxicity in NC	[95]
SWNTs	CD133 mAB	Recognise and specifically target the GNM-CD33^+^ cells	Glioblastoma	N/A	[96]
SWNTs	IGF1R mAB and HER2 mAB	Target IGF1R and HER2 surface receptors	Breast cancer	Negligible toxicity in NCs	[97]
SWNTs	RGD peptide	Target α_v_β_3_-expressing cancer cells	MM	⬇ Toxicity in vitro	[98]
SWNTs	EGF receptor	Active targeting ability and ⬆ uptake of drugs	HC, NC	Negligible toxicity	[99]

DOX = Doxorubicin; Cis = cisplatin; PEG = polyethylene glycol; FA = folic acid; MnO_2_ = manganese dioxide: Ce6 = Chlorin e6; CS/PNIPAAm = chitosan-poly (N-isopropyl acrylamide; TME = tumour microenvironment; DDDS = dual-drug delivery system; MRI = magnetic resonance image; AE = acidic environment; ⬆ = improving, improved, augmented, high, higher; ⬇ = low, lower, decreased; reduced; HA = hyaluronic acid: PA = particles aggregation; NTs = normal tissues; PC = positive charge; NCs = normal cells; PLGA = polylactic-co-glycolic acid; CD33 = cluster of differentiation-133; IGF1R = insulin-like growth factor 1 receptor; HER2 = human endothelial receptor 2; RGD = arginyl-glycyl-aspartic acid; MM = malignant melanoma; HC = head carcinoma; NC = neck carcinoma; WD = water dispersibility; WS = water solubility; SS = serum stability; MDR = multi-drug resistance; N/A = not acquired; TM = tumour model.

**Table 7:** Application of modified CNTs in anticancer photothermal therapy (PTT).

CNTs	Functional Molecules	Effectiveness	Tumour Model	Observations	Refs.
MWCNT	Ag NPs	⬆ NIR LA at 670 nm	Melanoma B16/F10 cells	⬆ CNT/Ag optical absorption to CNTs and Ag NPs	[210]
MWCNT	PEG	⬆ MWCNTs WS	Melanoma	⬇⬇⬇ Tumour size in the cancerous mice	[83]
MWCNT	–COO, –COOPt, –Pt NPs	–COO ⬆ CNTs WS, Pt NPs ⬆ LA at 1064nm	Cancer PC3 cells	PP ⬆ cytotoxicity due to ⬆ temperature of CCs	[190]
MWCNT	–COO, –COOAu, –Au NPs	–COO ⬆ CNTs WS, Au NPs ⬆ LA at 1064nm	MCF7 cells	PP ⬆ cytotoxicity due to ⬆ temperature of CCs	[189]
MWCNT	MTX, PEI, FA	MTX-based ACA, FA-based TP PEI-based hydrophilicity	MCF7 cells	⬆ Synergistic effect of MTX and PTT (808 nm laser) MWCNT-MTX-PEI-FA are absorbed selectively	[202]
MWCNT	PS MAA	Antibody against prostate-specific MA	Prostate cancer LNCaP cells	> 80% cell ablation with a single 30-s (a 532-nm)	[211]
MWCNT	Au NSs	⬆ Biocompatibility, ⬆ photothermal efficiency	Melanoma B16F10 mice	MWCNT/Au NSs have 12.4% ⬆ PE (808 nm)	[184]
MWCNT	PEG	⬆ Photothermal transduction (NIR)	BPH	⬇ Prostate size, apoptosis in PEC	[212]
SWCNTs/MWCNTs	PL, SL	BDS with ⬆ PE, TS	HeLa, MRC-5 cells	Muted surviving expression, ⬇⬇⬇ TG PE under 808 nm NIR light	[156]
MWCNT	TiO_2_ NPs	Absorb and scatter the 808 nm NIR light	Melanoma	Selective tumour death, ⬇ HR, ⬆ necrosis	[213]
SWCNT	anti-IGF-1R, CY7	WS, BS, LT SWCNT-CY7-IGF-1R	PDCA	Target to PDCA for dye imaging-guided PTT	[157]

⬇⬇⬇ = highly lowered, suppression; ⬆ = improving, improved, augmented, high, higher; ⬇ = low, lower, decreased; reduced; WS = water solubility; PEG = polyethylene glycol; LA = laser absorption; MTX = methotrexate; PEI =polyethylene amine; FA = folic acid; PS MAA = prostate-specific membrane antigen antibody; Au NSs = gold nano stars; PL = peptide lipid; SL = sucrose laurate; anti-IGF-1R = insulin-like growth factor receptor anti-IGF-1R antibody; CY7 = imaging agent; TiO_2_ NPs = titanium oxide nanoparticles; WS = water soluble; BS = bio stable; LT = low toxic; BDS = bifunctional delivery system; PDAC = pancreatic ductal adenocarcinoma; PE = photothermal effects; TS = temperature sensitivity; BPH = benign prostatic hyperplasia; PEC = prostatic epithelial cells; PTT = photothermal therapy; PP = plasmon phenomenon; CC = cancer cells; ACA = anticancer activity; TP = targeting power; TG = tumour growth; HR = heat resistance; BPH = benign prostatic hyperplasia.

**Table 8:** Application of modified CNTs in anticancer photodynamic therapy (PDT).

CNTs	Functionalized Molecules	Effectiveness	Tumour Model	Biocompatibility Test	Refs.
SWCNT	HA, Ce6	⬆ SWCNTs stability, WD (HA), Ce6-based ACA by PDT	Colon cancer cells	Colon cancer cell death, ⬆ PDT capacity	[194]
SWCNT	ZnMCPP	>50% ⬆ singlet and triplet oxygen quantum yields	MCF-7 breast cells	64% ⬇ CV at 40 M (C1), 97-95% ⬇ CV (C2,3)	[192]
MWCNT	OEG, DOX, FA	OEG used to link DOX with CNT DOX used as anticancer drug FA act as the system’s targeted ligand	HeLa, L929, A549	⬆ Killing of cancer cells when exposed to light.	[214]
SWCNT	AA, ZnMCPP	⬆ Lifetimes, quantum yields, singlet oxygen quantum yields	MCF-7	77% cytotoxicity by combined therapy	[193]

ACA = anticancer activity; ⬆ = improving, improved, augmented, high, higher; ⬇ = low, lower, decreased; reduced, reduction; HA = Hyaluronic acid; Ce6 = chlorine e6; ZnMCPP = zinc mono carboxy phenoxy phthalocyanine; OEG = ethylene glycol oligomers; DOX = doxorubicin; FA = folic acid; AA = ascorbic acid; WD = water dispersity; C1, C2, and C3 = complexes 1, 2, and 3; CV = cell viability.

**Table 9:** Applications of CNT-based drug delivery systems for enhanced phototherapy.

CNTs	FM	Effectiveness ^§^	TM	Biocompatibility	NIR Laser	Refs.
MWCNTs	PVPy-S-PEG-FA, DOX	⬆ ATA, ⬆ WD, ⬆ BCT, ⬆ DL, ⬆ PC of CNTs by PVPy, pH-sensitive	HeLa	⬆ Biocompatibility	808	[198]
MWCNTs	ED	⬆ ATA, ⬆ AA of MTX, ⬆ CD by SE, MWCNT-ED-MTX = MWCNT-ED-FA *	MCF7	N.R.	808	[202]
MWCNTs	Ge, Le	⬆ AA by LH, ⬆ biocompatibility, ⬆ HDP, ⬆ PC	MCF-7	⬇ Danger to NC	808	[200]
MWCNTs	MTX, PEI, FA	⬆ ATA, ⬆ WD by PEI, ⬆ AA of MTX by SE with PPT	MCF-7	N.R.	808	[202]
SWCNTs	ANXA5	⬆ Stimulation of IS, AE by SWCNT-ANXA5 SE	EMT6 BCCs	N.R.	980	[185]
CNTs	MXene	⬇ CNT aggregation, DOX DL = 85.6%, ⬇⬇⬇ HeLa cells by PTT + DOX SE	HeLa	N.R.	650/808	[203]

FM = Functionalizing molecules; TM = tumour model; PVPy = poly-N-vinyl pyrrole; S = sulphide bridge; PEG = polyethylene glycol; ED = ethylenediamine; Ge = gemcitabine; Le = lentinan; FA = folic acid; MTX = methotrexate; PEI = polyethyleneimine; ANXA5 = annexin 5 is a phospholipase A2 and protein kinase C inhibitory protein with calcium channel activity and a potential role in cellular signal transduction, inflammation, growth and differentiation; MXene = tuneable family of 2D carbides and nitrides; ⬆ = enhancing, enhanced, improving, improved, augmented, high, higher; ⬇ = low, lower, decreased; reduced, reduction; BCT = blood circulation time; WD = water dispersibility; ATA = active targeting ability; BCCs = breast cancer cells; AA = anticancer activity; PC = photothermal characteristics; HDP = hydrophilicity; IS = immune system; DL = drug loading; * for anticancer effects; CD = cell death; SE = synergistic effects; LH = localised hypothermia; ^§^ = under NIR radiation; AE = abscopal effect; ⬇⬇⬇ = suppression; NCs = normal cells.

**Table 10:** Application of modified CNTs in combined PTT and PDT for cancer treatment.

CNTs	FM	Effectiveness	TM	Biocompatibility	Refs.
SWCNHs	ICG	⬆ WD, ⬆ photostability, ⬆ AA by PTT + PDT SE	4T1 BCCs *	⬆ Photostability, ⬆ biocompatibility	[204]
SWCNHs	Ce6, Gd^3+^	⬆ IS, ⬆ ATA, ⬆ TP, ⬆ ISR by PTT + PDT SE in AMMs	4T1	N.R.	[206]
SWCNHs	Hyp	⬆ WS ⬇ 4T1 growth by PDT + PTT SE	4T1 *	⬆ Photostability, ⬆ biocompatibility	[205]
MWCNT	MnO_2_, Ce6	O_2_ + H^+^ production by MnO_2_ ⬇ TM, ⬆ potential for IG PDT + PTT SE	HeLa *	⬆ Biocompatibility	[207]
SWCNT	PEG, Fe_3_O_4_, CQDs, DOX, sgc8c	⬆ AA by PTT + PDT + CT by SE	HeLa mice	⬆ Biocompatibility	[208]
CNP@SiO_2_	DOX	pH responsive drug delivery, ⬆ PTT, ⬆ CT, ⬇⬇⬇ TD	4T1 cells	N.R.	[215]
SWCNT	HA, HMME	⬆ WS, HMME = PDT agent, ⬆ AA by PDT + PTT SE	B16F10, MMCs	⬆ Biocompatibility	[196]

SWCNHs = single-walled carbon nanohorns; DOX = doxorubicin; CNP@SiO_2_ = silica oxide carbon nanoparticles; ICG = indocyanine green; Ce6 = chlorine e6 photosensitizer; Gd^3+^ = gadolinio; Hyp = hypericin; PEG = polyethylene glycol; CQDs = carbon quantum dots; DOX = doxorubicin; sgc8c = aptamer; m-THPC = m-tetrahydroxy phenyl chlorin; HA = hyaluronic acid; HMME = hematoporphyrin monomethyl ether; FMs = functionalizing molecules; TM = tumour model; TD = tumour development; ⬆ = enhancing, enhanced, improving, improved, augmented, high, higher; ⬇ = low, lower, decreased; reduced, reduction; WD = water dispersibility; ATA = active targeting ability; TP = tumour penetration; BCCs = breast cancer cells; AA = anticancer activity; ISR = immune system response; SE = synergistic effects; ⬇⬇⬇ = suppression; WS = water solubility; OCC = ovarian cancer cells; CT = chemotherapy; PS = photosensitizer; MMCs = mice melanoma cells; AMMs = advanced metastatic malignancies; * in vivo and in vitro experiments; IG = imaging guided; MRI = magnetic resonance imaging; N.R. = not reported.

**Table 11:** CNTs application in anticancer immunotherapy.

CNTs	FM	Effectiveness	TM	Biocompatibility	Refs.
MWCNTs	COOH	⬆ WS, ⬆ CSA, ⬆ CP, ⬆ MA	H22 HCCs	N.F.	[237]
MWCNTs	Rg3	Puffed ginseng, ACA, Apoptosis, ⬇ PD-L1 expression	TNBC	N.F.	[171]
SWCNTs	CpG	ACA in gliomas, ⬇ CCP, ⬇ invasion/migration	HCCsT116	⬆ Mice survival rate	[238]
MWCNTs	DOX/CpG	⬆ WD; no change in MWCNTs PS, ⬇ TG, ⬆ CD4^+^, ⬆ CD8^+^, ⬆ T cells	B16 MCs	Biocompatible to normal cells	[172]
SWCNTs	COOH/OVA	Primary immunisation/mice, ⬆ anti-ovalbumin antibody response	AMBCs	⬇ Toxicity, ⬇ inflammation	[239]
MWCNTs	PDAEMA	⬆ Design flexibility, ⬆ control on SARs, ⬆ SE, ⬆ CU	B16-F10	⬆ Biocompatibility	[240]

FM = functionalizing molecules; Rg3 = Ginsenoside Rg3; CpG = complex; DOX = doxorubicin; OVA = Ovalbumin; PDAEMA = poly(2-dimethylaminoethylmethacrylate); WS = water solubility; WD = water dispersibility; ⬆ = enhancing, enhanced, improving, improved, augmented, high, higher; ⬇ = low, lower, decreased; reduced, reduction; ACA = anticancer activity; PS = physical structure; SAR = structure activity relationships; HCCs = hepatocarcinoma cells; TNBCs = triple-negative breast cancer; HCCsT116 = human colon cancer cell line T116; MCs = melanoma cells; AMBCs = activated mouse B cells; TM = tumour model; CSA = component system activation; CP = cytokine production; MA = macrophage activation; CCP = cancer cell proliferation; TG = tumour growth; SE = silencing efficiency; CU = cellular uptake; N.F. = not found.

**Table 12:** Applications of CNTs in cancer diagnosis.

CNTs	DM	Effect	Refs.
SWCNTs	RI	Spots of RSs were visible in colon-26 cells after 5 days of administration of o-SWNTs-PEG	[251]
SWCNTs	RI	⬆ SERS-E suitable for labelling and fast RI of biological samples	[252]
SWCNTs	MRI	Excellent MRI functions for tumour diagnosis	[253]
MWCNTs	MRI	⬆ Longitudinal proton relaxation process, T1 enhanced MRI effect	[254]
MWCNTs	US	⬆ Contrast in ultrasonic imaging	[255]
MWCNTs	US	Strong, long-lived and ⬆-quality ultrasound signal after sonication treatment	[256]
SWCNTs	PAI	Clear and stable PA signals	[257]
MWCNTs	PAI	Precise target to the tumour site in vivo, ⬆ PA imaging effect	[258]
MWCNTs	RNI	Easy and direct SPECT/CT imaging	[259]
SWCNTs	RNI	⬆ Tumour tissue accumulation for the subsequent radionuclide imaging	[260]
SWCNTs	NIR-FI	⬆ Signal-to-noise performance, ⬆ specificity to ovarian tumour and tumor nodules	[261]
SWCNTs	NIR-FI	⬆ Resolution intravital tumour vessel images through the thick skin in live mice	[262]
MWCNTs	NBSs	⬆ Biosensing ability, wide linear range for detecting miR-21, ⬇⬇⬇ detection limit	[263]
SWCNTs	NBSs	Broad detection range, ⬇ detection limit, ⬆ specificity to only OPN in prostate cancer	[264]
MWCNTs	NBSs	Simple detection of CDK1, report the enzymatic activity of CDK1 for cancer diagnosis	[265]
MWCNTs	NBSs	⬆ Selectivity/sensitivity to HPV-18, early, rapid, easy, and accurate diagnosis of cervical cancer	[266]

RI = Raman imaging; NBSs = nano-biosensors; NIR-FI = near infrared fluorescence imaging; CNTs in cancer imaging; RNI = radionucleotide imaging; US = ultrasonography; PAI = photoacoustic imaging; MRI = magnetic resonance imaging; DM = diagnostic method; RS = Raman signal; SERS-E = surface-enhanced Raman scattering effect; SPECT = single photon emission computed tomography; CT = computed tomography; ⬆ = strong, enhancing, enhanced, improving, improved, augmented, high, higher; ⬇ = low, lower, decreased; reduced, reduction; ⬇⬇⬇ = very low.

**Table 13:** Carbon nanotube-based cancer detection techniques. Reproduced from Singh et al. [85].

CNTs	Cell Line/Biomarkers	Linear Range	LoD	Techniques	Ref.
SWCNTs	PSA	N.R.	250 pg/mL	Electrochemical	[288]
SWCNTs	PSA	0.4–40 pg/mL	4 pg/mL	Immune sensing	[289]
MWCNTs	AFP	0.02–2.0 ng/mL	8.0 pg/mL	Immune sensing	[290]
MWCNTs	CEA	0.5–15.0 and 15.0–200 ng/mL	0.01 ng/mL	Immune sensing	[291]
MWCNTs	AFP	0.1–15.0 and 15.0–200.0 ng/mL	0.08 ng/mL	Immune sensing	[292]
MWCNTs	CA 19-9	12.5–270.0 U/mL	8.3 U/mL	Immune sensing	[293]
MWCNTs	hCG	Up to 600 mIU/mL	14.6 mIU/mL	Electrochemical	[294]
MWCNTs	hCG	0.8–500 mIU/mL	0.3 mIU/mL	Electrochemical	[295]
CNTs	PSA	1–100 ng/mL	1.0 ng/mL	Electrochemical	[296]
MWCNTs	CA 125	1.0–30 and 30–150 U/mL	0.36 U/mL	Electrochemical	[297]
CNTs	AFP	1–55 ng/mL	0.6 ng/mL	Immune sensing	[298]
MWCNTs	CA19-9	0–1000 U/mL	N.R.	Electrochemical	[299]
CNTs	GP73	0–80 ng/mL	58.1 pg/mL	Immune sensing	[300]
CNTs	AFP	0–64 ng/mL	47.1 pg/mL	Immune sensing	[300]
CNTs	AKT2 gene	1 pM–1 μM	2 fM	Electrochemical	[301]
CNTs	CA 125	0.001–0.1 ng/mL/0.1–30 ng/mL	0.5 pg/mL	Electrochemical	[302]
CNTs	Cyfra 21-1	0.1–10,000 ng/mL	0.5 ng/mL	Fluorescence	[303]
CNTs	HepG2	10–10^5^ cells/mL	5 cells/mL	Electrochemical	[304]

N.R. = Not reported.

**Table 14:** Clinical trials assessing the possible application of CNTs in anticancer therapy.

Trial Number	Sponsor	Device/Intervention	Problem	Participants	Study Status	Ending Year	Ref
NCT01773850 *	UNC Lineberger Comprehensive Cancer Center	CNT x-ray source array for SDT of BN	BN	54	Completed	2018	[315]
NCT01420588 **	Anhui Medical University	Of-AUNPs/CNT-based chemical nano sensors	PCGLs	1000	Completed	2020	[316,317]

SDT = stationary digital tomosynthesis; BN = breast neoplasm; PCGLs = pre-cancerous gastric lesions; AuNPs = gold nanoparticles; CNTs = carbon nanotubes: Of = organically functionalized; * study title: Stationary carbon nanotube X-ray digital breast tomosynthesis scanner; ** study title: Diagnosis of gastric lesions from exhaled breath and saliva.

**Table 15:** Study type assessment.

Trial Number	Sponsor	Study Type	Ethics Approval	Status	Ref
NCT01773850 *	UNC Lineberger Comprehensive Cancer Center	OCPS	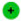	Completed	[315]
NCT01420588 **	Anhui Medical University	OCPS	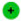	Completed	[316,317]

OCPS = Observational cohort prospective study * study title: Stationary carbon nanotube X-ray digital breast tomosynthesis scanner; ** study title: Diagnosis of gastric lesions from exhaled breath and saliva; 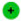 = positive ethical approval.

**Table 16:** Risk of bias in randomised studies as assessed by the Cochrane Collaboration’s “Risk of Bias” tool.

Trial Number	RSG	AC	BPP	BOA	IOD	SR	Ref
NCT01773850	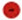	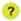	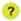	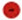	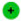	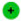	[315]
NCT01420588	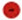	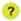	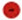	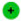	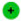	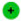	[316,317]

RSG = random sequence generation; AC = allocation concealment; BPP = blinding of participants and personnel; BOA = blinding of outcomes assessment; IOD = incomplete outcome data; SR = selective reporting.

**Table 17:** Tactics for diminishing toxicity of CNTs.

Strategy	Goal	Modifying Agents/Methods	Results	Refs
CNTs surface modification with biocompatible materials or other molecules	⬆ Dispersion in biological fluids Influenced CU, ⬆ Solubility ⬇ Toxicity	Proteins, surfactants	⬆ TT, ⬆ TB, ⬇ Toxicity	[341,342,343]
		FA	⬆ In vivo tumour targeting, ⬆ Therapeutic benefits ⬇ Toxicity	[141]
		PA hydrogels *, biomaterial, TiO_2_	100% survival of L929 mouse fibroblast	[339]
Coatings of CNTs	⬆ CNTs biocompatibility ⬇ Potential toxicity Prevent direct contact with BS ⬆ CNTs solubility	Curcumin lysine **	⬇ IL-6, IL-8, IL-1β, TNFα, N-FκB ⬆ Antioxidant enzyme catalase, ⬇ ROS generation Recovery of MM, ⬇ Cell death	[344]
CNTs encapsulation CNTs to entrap BAM	⬇ Direct cells exposure to CNTs Control of CNTs release ⬇ CNTs impact on tissues	PEG (entrapping agent) Oxaliplatin (entrapped agent)	PEGylation delayed oxaliplatin release rate ⬆ Drug’s anticancer effects on HT-29 cells	[112]
Tailor Ø size and L	⬇ Toxicity	N.A.	⬆ SSA, ⬆ TM, ⬇ Toxicity, ⬇ Harm to lysosomes ***	[345,346]
Optimized PP	Remove MI Remove RC	Chemical/electrochemical oxidation [347] High chlorine partial pressure [348] MA digestion [349] Incandescent annealing [350]	⬇ Lower harmful effects	[350]
Engineering controls Suitable PPE	⬇ Inhalation	Proper ventilation/respiratory protection	⬇ Respiratory toxicity	N.R.
CA with AO	⬇ OS ⬇ Damage to cells	Quercetin	Prevention of the oxidative damage ⬇ Inflammatory effects, ⬇ Immuno-toxic effects	[336]

* Encapsulation agent for CNTs-COOH; ** used to coat MWCNTs; N.A. = not applicable; N.R. = not reported; ⬇ indicates minor reduction, lower, decreased, decrease; ⬆ indicates improved, increase, increased, major; PPE = personal protective equipment; OS = oxidative stress; BAM = bioactive molecules; Ø = diameter; L = length; PP = purification processes; CA = co-administration; AO = antioxidants; CU = cellular uptake; MI = metal impurities; RC = residual catalysts; BS = biological systems; PA = polyacrylamide; FA = folic acid; MA = microwave assisted; TT = tumour targeting; TB = therapeutic benefits; SSA = specific surface area; TM = transmembrane mobility; MM = mitochondrial membrane; *** large Ø MWCNTs.

## Data Availability

No new data were created in this review article.

## Supplementary Materials

The following supporting information can be downloaded at: https://www.mdpi.com/article/10.3390/cells14141052/s1. Table S1. Structural properties of various modified/activated CNTs vs. pristine CNTs (first row) [351,352,353,354,355,356,357,358]. Table S2. Main methods to synthesise carbon nanotubes (CNTs) [54,55,56,358,359,360,361,362,363,364,365,366,367,368,369,370,371,372,373,374,375,376,377,378,379,380,381,382]. Table S3. Most relevant research articles on CNT synthesis from biomass by different methods, operating parameters, and main properties [382,383,384,385,386,387,388,389,390,391,392,393,394].
