# Supplementary material for: Carbon Nanotubes as Excellent Adjuvants for Anticancer Therapeutics and Cancer Diagnosis: A Plethora of Laboratory Studies Versus Few Clinical Trials
Source: Cells. 2025 Jul 9;14(14):1052. doi: 10.3390/cells14141052 (PMC12293991; doi:10.3390/cells14141052)
Supplement: Supplementary file 1 [file cells-14-01052-s001.zip › cells-3693917-supplementary.pdf]

Supplementary Materials

Carbon Nanotubes as Excellent Adjuvants for Anticancer Therapeutics and Cancer Diagnosis: A Plethora of Laboratory Studies versus Few Clinical Trials

Silvana Alfei [1,*], Caterina Reggio [2] and Guendalina Zuccari [1,2,*]

[1] Department of Pharmacy (DIFAR), University of Genoa, Viale Cembrano, 4, 16148 Genoa, Italy
[2] Laboratory of Experimental Therapies in Oncology, IRCCS Istituto Giannina Gaslini, Via G. Gaslini 5, 16147 Genoa, Italy; caterinareggio@gaslini.org (C.T.)
* Correspondence: alfei@difar.unige.it; Tel.: +39 010 355 2296 (S.A.); guendalina.zuccari@unige.it (G.Z.)

Tables

The list of references reported in Tables is available under the last Table S3.

Table S1. Structural properties of various modified/activated CNTs vs pristine CNTs (first row).

CNTs	BET (m²/g)	MIV (cm³/g)	MEV (cm³/g)	Characteristics	Refs.
Pristine	150–1587	0.06–0.15	0.85	N.A.P.	[1–6]
HNO_3 modified	157	N.A.	0.37	AT provides O_2 containing -COOH, -OH	[7]
NH_3 modified	195	N.A.	0.42	NH_3 removes -COOH, -OH	[7]
KOH modified	785	0.17	1.04	KOH ↑ micropores and mesopores<br>↑ KOH/CNT ratio, ↑ PV	[3–6]
Air activated	270	0.06	0.56	↓ Micropore volume<br>Air removes catalyst metals/AC	[3]
CO_2 activated	420	0.10	0.67	Large micropore volume	[3]
O_3 treated	320	0.12	0.69	Opens end caps<br>Holes in sidewalls<br>OOCA, O_2 containing functional groups	[2]
HY	550	0.18	0.97	↑ External surface area	[8]

N.A. = Not available; N.A.P. = not applicable; HT = heat treatment; AT = acid treatment; PV = pore volume; AC = amorphous carbon; OOCA = ozonolysis oxidised carbon atoms; ↑ denotes high, higher, improved, enhanced; ↓ denotes reduced.

Table S2. Main methods to synthesize carbon nanotubes (CNTs).

Method	Invention paternity/Description	Tube type	Advantages/Disadvantages	Refs
AD	(1991) From procedure to get fullerenes applying a current (110 A) at T >1,700°C<br>CNTs form in the carbon soot of GR electrode	N.R.	⬇Structural defects<br>Macroscopic production	[9–11]
LA	Dr. Richard Smalley *<br>Graphite is blasted with a laser<br>Graphite and cobalt/nickel (metal catalyst particles) blasted with a laser	MWCNTs<br>SWCNTs	N.R.	[12,13]<br>[14]<br>[15]
CVD	Substrate of nickel, cobalt, iron, or combination catalyst NPs<br>Heating at 700°C under a flux of a “process gas” ** and a “carbon-containing gas” *** to promote CNTs growing<br>CNTs directly growth on the desired substrate by careful deposition of the catalyst	N.R.	⬇ Cost, scaling up<br>Industrial production<br>If present, need to remove the catalyst support via acid treatment, with possible damage to the CNT structure	[16–21]<br>[16,22]
PECVD	Advanced CVD consisting of plasma generation by applying a strong electric field during CNTs growth	VACNTs	N.R.	[23]
HiPco	Rice University<br>HPCO reacts with FePC, Fe NPs form providing the nucleation surface where CNTs form by the transformation of CO into carbon	SWCNTs	Production from milligrams to grams scale No environmental release of wastes	[12,13]
SGCVD	Kenji Hata, Sumio Iijima at AIST (Japan)<br>Introduction of water into the CVD reactor	VANTAs<br>Forest material	⬆Activity and lifetime of the catalyst<br>⬆To LA and HiPco by 100 times<br>⬇ Mass density, >99.98% pure, 2.5 mm height SWNT 10min<br>Easy separation of CNTs from the catalyst<br>>1 mm long VANTAs in several shapes §	[24]<br>[13,25]
PT	Olivier Smiljanic (2000), Institut National de la Recherche Scientifique (INRS), Varennes (Canada)<br>Argon, ethylene and ferrocene into PLASMOTRON, thus developing an intense 'flame' containing CNTs, metallic, carbon NPs and amorphous carbon	SWCNTs	10-fold ⬇consumed energy than in LA or AD.	[13,26]
ITP	Sherbrooke University and the National Research Council of Canada<br>Modified PT procedure<br>TP is generated by HFOCs in a loop and is conserved in flowing inert gas	SWCNTs	CNTs with different diameter distributions	[27]
LEM	Metal ions reduced to metal forms on cathode provide the nucleation surface for CNTs growth which derive from electrolysis of molten carbonates #	MWCNTs	⬆Valued CNTs<br>Strategy for CO_2 capture and conversions	[13,28,29]
NICFE	CNTs form naturally in flames emitted by burning methane, ethylene, benzene		⬆Irregular in dimensions and quality CNT	[30–32]
ACVD	CNTs are synthesized in the gas phase and deposited in the form of randomly oriented networks, ready for many applications including transparent conductors.	SWCNTs TF	Clean defect less SWCNTs with limited yield	[33–35]

PR = Parameters; Adv = advantages; Disadv = disadvantages; * Rice University; AD = arc discharge; LA = laser ablation; CVD 0 chemical vapor deposition; ⬇ = less, minor, low; ** ammonia, nitrogen, or hydrogen; *** acetylene, ethylene, ethanol, or methane; PECVD = plasma-enhanced chemical vapor deposition; VACNTs = vertically aligned CNTs; HiPco = High-Pressure Carbon Monoxide Process; HPCO = high-pressure carbon monoxide;

FePC = iron pentacarbonyl; SGCVD = super-growth CVD, also known as water-assisted chemical vapor deposition; VANTAs = millimetre-high vertically aligned nanotube arrays; "forests" materials = tubes aligned to the substrate; ACVD = aerosol CVD; TF = thin films; ⬆ = enhanced, improved, high, higher, highly, superior; § = sheets and bars, by applying weak compression during the process; PT = plasma torch; PLASMOTRON = microwave plasma torch; LEM = liquid electrolysis method; NICFE = natural, incidental, and controlled flame environments; GR = graphene; HFOCs = high-frequency oscillating currents; TP = thermal plasma; # the reactant is a carbon dioxide greenhouse gas.

Table S3. Most relevant research articles on CNT synthesis from biomass by different methods, operating parameters and main properties.

Feed stock	Method	Support/Catalyst	Operating parameters			CNTs	Properties				Refs
			T (°C)	Time (min)	Carrier gas		SA (m^2/g)	D (nm)	*(%)	L (μm)	
Rice straw	P	Al_2O_3/Fe-Ni	830	30	N_2	MWCNT	188	15–40	N.A./N.A.	N.A.	[36]
Olive oil	P	Si wafer/$NiCl_2$	900	60	Ar	SWCNT	N.A.	27–31	N.A./ N.A.	N.A.	[37]
Turpentine oil	P	Zeolite/Fe-Co	850	25	N_2	SWCNT	N.A.	7–20	58/N.A.	N.A.	[38]
Coconut oil	CVD	Fe	850	60	N_2	MWCNT	N.A.	80–100	58/N.A.	3–4	[39]
Sesame oil	P+CVD	FNS/CH_3CN	900	15	Ar	MWCNT	N.A.	30–60	58/N.A.	3–4	[40]
Palm oil	CVD	Silicon/Ferrocene	750	30	Ar	SWCNT<br>MWCNT	N.A.	0.6–1.2	90/N.A.	110	[41]
Plastic waste	P+CVD	Ni	800	30	N_2	MWCNT	N.A.	40–50	N.A./31	N.A.	[42]
Petroleum coke	CVD	Silica/Fe	700	60	He	MWCNT	N.A.	18	N.A./N.A.	N.A.	[43]
Plastic waste	P	Cordierite/*N*-Mg	750	60	N_2	MWCNT	N.A.	30–50	N.A./93	30–50	[44]
NCG	P+CVD	Ni/Al_2O_3	1000	112	N_2	SWCNT	N.A.	10	N.A./82	N.A.	[45]
Ethylene	FC, CVD	Co/Fe, Co/Ni, Ni, Co, Fe	1050	0.17	N_2	SWCNT	N.A.	0.67–2	61–69/ N.A.	N.A.	[46]
Rice straw	T-CVD	Fe, Ni	800	120	N_2	MWCNT	20<br>(CNT+Fe)35<br>(CNT+Fe+Ni)	22<br>(CNT+Fe)66<br>(CNT+Fe+Ni)	N.A./41–44	N.A.	[47]

*Purity/yield; CH_3CN = acetonitrile; N.A. = information not available; D = Diameter; P = pyrolysis; SA = surface area; T = temperature; NCG = natural condensed gas; floating catalyst; CVD = chemical vapor deposition; T-CVD = thermal CVD; FNS = ferrocene nitrogen source.

References

1. Agnihotri, S.; Mota, J.P.B.; Rostam-Abadi, M.; Rood, M.J. Structural Characterization of Single-Walled Carbon Nanotube Bundles by Experiment and Molecular Simulation. *Langmuir* **2005**, *21*, 896–904, doi:10.1021/la047662c.
2. Hemraj-Benny, T.; Bandosz, T.J.; Wong, S.S. Effect of Ozonolysis on the Pore Structure, Surface Chemistry, and Bundling of Single-Walled Carbon Nanotubes. *J. Colloid Interface Sci.* **2008**, *317*, 375–382, doi:10.1016/j.jcis.2007.09.064.
3. Chen, Y.; Liu, C.; Li, F.; Cheng, H.-M. Pore Structures of Multi-Walled Carbon Nanotubes Activated by Air, CO2 and KOH. *J. Porous. Mater.* **2006**, *13*, 141–146, doi:10.1007/s10934-006-7017-6.
4. Lee, S.M.; Lee, S.C.; Jung, J.H.; Kim, H.J. Pore Characterization of Multi-Walled Carbon Nanotubes Modified by KOH. *Chem. Phys. Lett.* **2005**, *416*, 251–255, doi:10.1016/j.cplett.2005.09.107.
5. Liu, Y.; Shen, Z.; Yokogawa, K. Investigation of Preparation and Structures of Activated Carbon Nanotubes. *Mater. Res. Bull.* **2006**, *41*, 1503–1512, doi:10.1016/j.materresbull.2006.01.017.
6. Niu, J.J.; Wang, J.N.; Jiang, Y.; Su, L.F.; Ma, J. An Approach to Carbon Nanotubes with High Surface Area and Large Pore Volume. *Microporous and Mesoporous Mater.* **2007**, *100*, 1–5, doi:10.1016/j.micromeso.2006.10.009.
7. Liao, Q.; Sun, J.; Gao, L. Adsorption of Chlorophenols by Multi-Walled Carbon Nanotubes Treated with HNO3 and NH3. *Carbon N. Y.* **2008**, *46*, 553–555, doi:10.1016/j.carbon.2007.12.009.
8. LU, C.; SU, F. Adsorption of Natural Organic Matter by Carbon Nanotubes. *Sep. Purif. Technol.* **2007**, *58*, 113–121, doi:10.1016/j.seppur.2007.07.036.
9. Iijima, S. Helical Microtubules of Graphitic Carbon. *Nature* **1991**, *354*, 56–58, doi:10.1038/354056a0.
10. Ebbesen, T.W.; Ajayan, P.M. Large-Scale Synthesis of Carbon Nanotubes. *Nature* **1992**, *358*, 220–222, doi:10.1038/358220a0.
11. Eatemadi, A.; Daraee, H.; Karimkhanloo, H.; Kouhi, M.; Zarghami, N.; Akbarzadeh, A.; Abasi, M.; Hanifehpour, Y.; Joo, S.W. Carbon Nanotubes: Properties, Synthesis, Purification, and Medical Applications. *Nanoscale Res. Lett.* **2014**, *9*, 393, doi:10.1186/1556-276X-9-393.
12. Alfei, S.; Schito, G.C. Antimicrobial Nanotubes: From Synthesis and Promising Antimicrobial Upshots to Unanticipated Toxicities, Strategies to Limit Them, and Regulatory Issues. *Nanomaterials* **2025**, *15*, 633. https://doi.org/10.3390/nano15080633.
13. Alfei, S.; Schito, G.C. Nanotubes: Carbon-Based Fibers and Bacterial Nano-Conduits Both Arousing a Global Interest and Conflicting Opinions. *Fibers* **2022**, *10*, 75. https://doi.org/10.3390/fib10090075.
14. Guo, T.; Nikolaev, P.; Rinzler, A.G.; Tomanek, D.; Colbert, D.T.; Smalley, R.E. Self-Assembly of Tubular Fullerenes. *J Phys. Chem.* **1995**, *99*, 10694–10697, doi:10.1021/j100027a002.
15. Guo, T.; Nikolaev, P.; Thess, A.; Colbert, D.T.; Smalley, R.E. Catalytic Growth of Single-Walled Nanotubes by Laser Vaporization. *Chem. Phys. Lett.* **1995**, *243*, 49–54, doi:10.1016/0009-2614(95)00825-O.
16. Kumar, M.; Ando, Y. Chemical Vapor Deposition of Carbon Nanotubes: A Review on Growth Mechanism and Mass Production. *.J Nanosci. Nanotechnol.* **2010**, *10*, 3739–3758, doi:10.1166/jnn.2010.2939.
17. Neupane, S.; Lastres, M.; Chiarella, M.; Li, W.; Su, Q.; Du, G. Synthesis and Field Emission Properties of Vertically Aligned Carbon Nanotube Arrays on Copper. *Carbon N. Y.* **2012**, *50*, 2641–2650, doi:10.1016/j.carbon.2012.02.024.
18. Inami, N.; Mohamed, M.A.; Shikoh, E.; Fujiwara, A. Synthesis-Condition Dependence of Carbon Nanotube Growth by Alcohol Catalytic Chemical Vapor Deposition Method. *Sci. Technol. Adv. Mater.* **2007**, *8*, 292–295, doi:10.1016/j.stam.2007.02.009.
19. Ishigami, N.; Ago, H.; Imamoto, K.; Tsuji, M.; Iakoubovskii, K.; Minami, N. Crystal Plane Dependent Growth of Aligned Single-Walled Carbon Nanotubes on Sapphire. *J. Am. Chem. Soc.* **2008**, *130*, 9918–9924, doi:10.1021/ja8024752.
20. Naha, S.; Puri, I.K. A Model for Catalytic Growth of Carbon Nanotubes. *J. Phys. D. Appl. Phys.* **2008**, *41*, 065304, doi:10.1088/0022-3727/41/6/065304.
21. Banerjee, S.; Naha, S.; Puri, I.K. Molecular Simulation of the Carbon Nanotube Growth Mode during Catalytic Synthesis. *Appl. Phys. Lett.* **2008**, *92*, 233121, doi:10.1063/1.2945798.
22. Eftekhari, A.; Jafarkhani, P.; Moztarzadeh, F. High-Yield Synthesis of Carbon Nanotubes Using a Water-Soluble Catalyst Support in Catalytic Chemical Vapor Deposition. *Carbon N. Y.* **2006**, *44*, 1343–1345, doi:10.1016/j.carbon.2005.12.006.
23. Ren, Z.F.; Huang, Z.P.; Xu, J.W.; Wang, J.H.; Bush, P.; Siegal, M.P.; Provencio, P.N. Synthesis of Large Arrays of Well-Aligned Carbon Nanotubes on Glass. *Science (1979)* **1998**, *282*, 1105–1107, doi:10.1126/science.282.5391.1105.

24. Futaba, D.N.; Hata, K.; Yamada, T.; Hiraoka, T.; Hayamizu, Y.; Kakudate, Y.; Tanaike, O.; Hatori, H.; Yumura, M.; Iijima, S. Shape-Engineerable and Highly Densely Packed Single-Walled Carbon Nanotubes and Their Application as Super-Capacitor Electrodes. *Nat. Mater.* **2006**, *5*, 987–994, doi:10.1038/nmat1782.
25. Hata, K.; Futaba, D.N.; Mizuno, K.; Namai, T.; Yumura, M.; Iijima, S. Water-Assisted Highly Efficient Synthesis of Impurity-Free Single-Walled Carbon Nanotubes. *Science (1979)* **2004**, *306*, 1362–1364, doi:10.1126/science.1104962.
26. Smiljanic, O.; Stansfield, B.L.; Dodelet, J.-P.; Serventi, A.; Désilets, S. Gas-Phase Synthesis of SWNT by an Atmospheric Pressure Plasma Jet. *Chem. Phys. Lett.* **2002**, *356*, 189–193, doi:10.1016/S0009-2614(02)00132-X.
27. Kim, K.S.; Cota-Sanchez, G.; Kingston, C.T.; Imris, M.; Simard, B.; Soucy, G. Large-Scale Production of Single-Walled Carbon Nanotubes by Induction Thermal Plasma. *J. Phys. D. Appl. Phys.* **2007**, *40*, 2375–2387, doi:10.1088/0022-3727/40/8/S17.
28. Ren, J.; Li, F.-F.; Lau, J.; González-Urbina, L.; Licht, S. One-Pot Synthesis of Carbon Nanofibers from CO_2. *Nano Lett.* **2015**, *15*, 6142–6148, doi:10.1021/acs.nanolett.5b02427.
29. Service, R.F. Conjuring Chemical Cornucopias out of Thin Air. *Science (1979)* **2015**, *349*, 1160–1160, doi:10.1126/science.349.6253.1160.
30. Yuan, L.; Saito, K.; Pan, C.; Williams, F.A.; Gordon, A.S. Nanotubes from Methane Flames. *Chem. Phys. Lett.* **2001**, *340*, 237–241, doi:10.1016/S0009-2614(01)00435-3.
31. Yuan, L.; Saito, K.; Hu, W.; Chen, Z. Ethylene Flame Synthesis of Well-Aligned Multi-Walled Carbon Nanotubes. *Chem. Phys. Lett.* **2001**, *346*, 23–28, doi:10.1016/S0009-2614(01)00959-9.
32. Duan, H.M.; McKinnon, J.T. Nanoclusters Produced in Flames. *J. Phys. Chem.* **1994**, *98*, 12815–12818, doi:10.1021/j100100a001.
33. Novikov, I. V.; Krasnikov, D. V.; Lee, I.H.; Agafonova, E.E.; Serebrennikova, S.I.; Lee, Y.; Kim, S.; Nam, J.; Kondrashov, V.A.; Han, J.; et al. Aerosol CVD Carbon Nanotube Thin Films: From Synthesis to Advanced Applications: A Comprehensive Review. *Adv. Mater.* **2025**, *37*, 2413777 doi:10.1002/adma.202413777.
34. Khabushev, E.M.; Kolodiazhnaia, J. V.; Krasnikov, D. V.; Nasibulin, A.G. Activation of Catalyst Particles for Single-Walled Carbon Nanotube Synthesis. *Chem. Eng. J.* **2021**, *413*, 127475, doi:10.1016/J.CEJ.2020.127475.
35. Nasibulin, A.G.; Moisala, A.; Brown, D.P.; Jiang, H.; Kauppinen, E.I. A Novel Aerosol Method for Single Walled Carbon Nanotube Synthesis. *Chem. Phys. Lett.* **2005**, *402*, 227–232, doi:10.1016/J.CPLETT.2004.12.040.
36. Lotfy, V.F.; Fathy, N.A.; Basta, A.H. Novel Approach for Synthesizing Different Shapes of Carbon Nanotubes from Rice Straw Residue. *J. Environ. Chem. Eng.* **2018**, *6*, 6263-6274, doi:10.1016/j.jece.2018.09.055.
37. Hamid, Z.A.; Azim, A.A.; Mouez, F.A.; Rehim, S.S.A. Challenges on Synthesis of Carbon Nanotubes from Environmentally Friendly Green Oil Using Pyrolysis Technique. *J. Anal. Appl. Pyrolysis* **2017**, *126*, 218-229, doi:10.1016/j.jaap.2017.06.005.
38. Ghosh, P.; Soga, T.; Afre, R.A.; Jimbo, T. Simplified Synthesis of Single-Walled Carbon Nanotubes from a Botanical Hydrocarbon: Turpentine Oil. *J. Alloys. Compd.* **2008**, *462*, 289-293, doi:10.1016/j.jallcom.2007.08.027.
39. Paul, S.; Samdarshi, S.K. A Green Precursor for Carbon Nanotube Synthesis. *New Carbon Mater.* **2011**, *26*, 85-88, doi:10.1016/S1872-5805(11)60068-1.
40. Kumar, R.; Singh, R.K.; Tiwari, R.S. Growth Analysis and High-Yield Synthesis of Aligned-Stacked Branched Nitrogen-Doped Carbon Nanotubes Using Sesame Oil as a Natural Botanical Hydrocarbon Precursor. *Mater. Des.* **2016**, *94*, 166-175, doi:10.1016/j.matdes.2016.01.025.
41. Suriani, A.B.; Azira, A.A.; Nik, S.F.; Md Nor, R.; Rusop, M. Synthesis of Vertically Aligned Carbon Nanotubes Using Natural Palm Oil as Carbon Precursor. *Mater. Lett.* **2009**, *63*, 2704-2706, doi:10.1016/j.matlet.2009.09.048.
42. Moo, J.G.S.; Veksha, A.; Oh, W. Da; Giannis, A.; Udayanga, W.D.C.; Lin, S.X.; Ge, L.; Lisak, G. Plastic Derived Carbon Nanotubes for Electrocatalytic Oxygen Reduction Reaction: Effects of Plastic Feedstock and Synthesis Temperature. *Electrochem. Commun.* **2019**, *101*, 11-18, doi:10.1016/j.elecom.2019.02.014.
43. Abdullayeva, S.H.; Musayeva, N.N.; Jabbarov, R.B.; Matsuda, T. Synthesis of Carbon Nanotubes from Byproducts of Oil Refiner. *WJCMP* **2014**, *04*, 93-100, doi:10.4236/wjcmp.2014.43014.
44. Wang, J.; Shen, B.; Lan, M.; Kang, D.; Wu, C. Carbon Nanotubes (CNTs) Production from Catalytic Pyrolysis of Waste Plastics: The Influence of Catalyst and Reaction Pressure. *Catal Today* 2020, *351*, 50-57, doi:10.1016/j.cattod.2019.01.058
45. Boufades, D.; Hammadou Née Mesdour, S.; Moussiden, A.; Benmebrouka, H.; Ghouti, M.; Kaddour, O. Optimization of Carbon Nanotubes Synthesis via Pyrolysis over Ni/Al2O3 Using Response Surface Methodology. *Fuller. Nanotub. Car. N.* **2022**, *30*, 467-475, doi:10.1080/1536383X.2021.1956475.

46. Ahmad, S.; Liao, Y.; Hussain, A.; Zhang, Q.; Ding, E.X.; Jiang, H.; Kauppinen, E.I. Systematic Investigation of the Catalyst Composition Effects on Single-Walled Carbon Nanotubes Synthesis in Floating-Catalyst CVD. *Carbon N. Y.* **2019**, *149*, 318-327, doi:10.1016/j.carbon.2019.04.026.
47. Fathy, N.A. Carbon Nanotubes Synthesis Using Carbonization of Pretreated Rice Straw through Chemical Vapor Deposition of Camphor. *RSC Adv.* **2017**, *7*, 28535-28541, doi:10.1039/c7ra04882c.

Disclaimer/Publisher's Note: The statements, opinions and data contained in all publications are solely those of the individual author(s) and contributor(s) and not of MDPI and/or the editor(s). MDPI and/or the editor(s) disclaim responsibility for any injury to people or property resulting from any ideas, methods, instructions or products referred to in the content.
